# An Investigation of Severe Influenza Cases in Russia during the 2022–2023 Epidemic Season and an Analysis of HA-D222G/N Polymorphism in Newly Emerged and Dominant Clade 6B.1A.5a.2a A(H1N1)pdm09 Viruses

**DOI:** 10.3390/pathogens13010001

**Published:** 2023-12-19

**Authors:** Natalia P. Kolosova, Nikita D. Boldyrev, Svetlana V. Svyatchenko, Alexey V. Danilenko, Natalia I. Goncharova, Kyunnei N. Shadrinova, Elena I. Danilenko, Galina S. Onkhonova, Maksim N. Kosenko, Maria E. Antonets, Ivan M. Susloparov, Tatiana N. Ilyicheva, Vasily Y. Marchenko, Alexander B. Ryzhikov

**Affiliations:** State Research Centre of Virology and Biotechnology “Vector”, Rospotrebnadzor, Koltsovo, Novosibirsk 630559, Russiashadrinova_kn@vector.nsc.ru (K.N.S.); danilenko_ei@vector.nsc.ru (E.I.D.); ilicheva_tn@vector.nsc.ru (T.N.I.);

**Keywords:** influenza, monitoring, A(H1N1)pdm09, D222G/N polymorphism, severe cases

## Abstract

In Russia, during the COVID-19 pandemic, a decrease in influenza circulation was initially observed. Influenza circulation re-emerged with the dominance of new clades of A(H3N2) viruses in 2021–2022 and A(H1N1)pdm09 viruses in 2022–2023. In this study, we aimed to characterize influenza viruses during the 2022–2023 season in Russia, as well as investigate A(H1N1)pdm09 HA-D222G/N polymorphism associated with increased disease severity. PCR testing of 780 clinical specimens showed 72.2% of them to be positive for A(H1N1)pdm09, 2.8% for A(H3N2), and 25% for influenza B viruses. The majority of A(H1N1)pdm09 viruses analyzed belonged to the newly emerged 6B.1A.5a.2a clade. The intra-sample predominance of HA-D222G/N virus variants was observed in 29% of the specimens from A(H1N1)pdm09 fatal cases. The D222N polymorphic variant was registered more frequently than D222G. All the B/Victoria viruses analyzed belonged to the V1A.3a.2 clade. Several identified A(H3N2) viruses belonged to one of the four subclades (2a.1b, 2a.3a.1, 2a.3b, 2b) within the 3C.2a1b.2a.2 group. The majority of antigenically characterized viruses bore similarities to the corresponding 2022–2023 NH vaccine strains. Only one influenza A(H1N1)pdm09 virus showed reduced inhibition by neuraminidase inhibitors. None of the influenza viruses analyzed had genetic markers of reduced susceptibility to baloxavir.

## 1. Introduction

Influenza is a highly contagious infectious disease in the form of local outbreaks, annual seasonal epidemics, and sometimes pandemics. In humans, influenza infection can cause severe disease and result in fatal outcomes, especially among individuals at high risk of influenza complications [1]. After its beginning in 2019, the COVID-19 pandemic considerably affected the global circulation of influenza viruses [2]. Following the severely limited flu activity in 2020–2021 [3], influenza virus circulation re-emerged during the 2021–2022 season with a predominance of the A(H3N2) viruses from the new genetic clade [4]. During the subsequent 2022–2023 flu season, influenza A(H1N1)pdm09 and B/Victoria viruses of new clades antigenically different from pre-pandemic variants had also spread globally [5]. During the 2022–2023 flu season, influenza A(H3N2) viruses remained predominant in the Northern Hemisphere (NH). They were outnumbered by B/Victoria viruses only toward the end of the season [6,7]. Throughout the entire season, SARS-CoV-2 viruses of the Omicron variant co-circulated with influenza viruses [8].

Epidemiological monitoring in Russia showed that the 2022–2023 flu season was more intense than the previous 2021–2022 season, and relatively more intense than all pre-pandemic flu seasons since 2016. Influenza A(H1N1)pdm09 viruses were the most prevalent in Russia in 2022–2023 followed by the B/Victoria viruses, which became predominant in circulation toward the end of the season [8].

Since their emergence in 2009, the evolution of A(H1N1)pdm09 viruses was accompanied by mutations in the hemagglutinin (HA) gene, leading to the emergence of new genetic clades, some of which were antigenically distinct.

In Russia, the most intense epidemics since 2015 were associated with the predominance of A(H1N1)pdm09 viruses [8]. One of the most important virulence markers of A(H1N1)pdm09 viruses is HA amino acid substitution (AAS) D222G/N, located in the receptor-binding site (RBS) and associated with increased disease severity and fatal outcomes [9,10,11,12,13]. D222G AAS was present in some of the sequenced 1918 pandemic strains [14]. HA-D222G/N substitutions were occasionally detected among seasonal H1N1 viruses circulated prior to the 2009 pandemic, as well as in A(H1N1)pdm09 pandemic viruses from their emergence in 2009 to the present [10,14,15]. In addition, a meta-analysis of available epidemiological evidence of A(H1N1)pdm09 virulence factors based on a comprehensive study of worldwide reported data for 2009–2012 showed an association for a single AAS D222G in HA with a significant increase in disease severity and fatality risk [9]. The increased pathogenicity of viruses with HA-D222G AAS was also demonstrated in an animal model [16,17,18,19]. The accumulation of D222G/N AAS may be altered by changes in the RBS. The new clades of influenza A(H1N1)pdm09 viruses that spread in Russia and other parts of the world during the 2022–2023 flu season were characterized by a subset of clade-specific AASs, some of which were located in the RBS (A186T, Q189E, E224A). This makes it important to assess the presence of HA-D222G/N polymorphism in recently circulating influenza A(H1N1)pdm09 viruses.

The monitoring of D222G/N polymorphism as a virulence marker of A(H1N1)pdm09 viruses may become an important element of influenza epidemiologic surveillance [15].

The continuous circulation of seasonal influenza viruses, leading to seasonal epidemics and substantial disease burden, fast diversification and evolution of influenza viruses, and ongoing cocirculation with SARS-CoV-2, requires thorough influenza virus monitoring. Such monitoring is required for the analysis of the genetic variability, antigenic properties, and drug susceptibility in order to enable the development and optimization of prevention and treatment strategies in response to influenza epidemics.

The aim of this work was to perform a genetic and virological analysis of influenza viruses detected in Russia in 2022–2023, including viruses from fatal influenza cases. An additional aim included an analysis of the D222G/N polymorphism in HA of A(H1N1)pdm09 strains. Results showed that influenza A(H1N1)pdm09 viruses from the 6B.1A.5a.2a clade (5a.2a) were predominant in circulation with a substantial circulation of influenza B viruses toward the end of the season and sporadic circulation of A(H3N2) viruses. This study showed a substantial frequency of D222G/N polymorphism among fatal cases of A(H1N1)pdm09 influenza, highlighting the need for continuous monitoring of the polymorphism.

## 2. Materials and Methods

### 2.1. Sample Preparation and Influenza Diagnostics

The study of clinical material (nasopharyngeal swabs) and frozen autopsy tissues (trachea, bronchi, or lungs) was conducted according to the guidelines of the Declaration of Helsinki and was approved by the Ethics Committee IRB 00001360 affiliated with the Federal Budgetary Research Institution State Research Center of Virology and Biotechnology “Vector”, Rospotrebnadzor (SRC VB “Vector”). Samples were collected at the local Sanitary-and-Epidemiological Centers of the Federal Service for Surveillance on Consumer Rights Protection and Human Wellbeing after obtaining written, informed consent from the patients or their close relatives in accordance with the regulations of the Russian Federation. PCR-based diagnostics of the original material for influenza virus RNA were conducted in local laboratories, and then all the positive samples were sent to the SRC VB “Vector”. All samples received at the SRC VB “Vector” were retested using diagnostic PCR [20]. Diagnostic real-time PCR for detecting influenza A and B viruses was performed using the reagent kits “AmpliSense Influenza virus A/B-FL”, “AmpliSense Influenza virus H1N1pdm2009-FL”, and “AmpliSense Influenza virus H3N2-FL” manufactured by the Central Research Institute of Epidemiology of the Federal Service for Surveillance on Consumer Rights Protection and Human Wellbeing (CRIE, Moscow, Russia). RNA was isolated with the “RIBO-prep” kit (CRIE, Moscow, Russia), and cDNA was synthesized using the “RevertaL” kit (CRIE, Moscow, Russia).

### 2.2. Sequence Analysis of Influenza Viruses

Sequencing was carried out at the SRC VB “Vector”, Rospotrebnadzor. To determine the nucleotide sequences of viral genes and genomes, viral RNA was isolated using the RIBO-sorb RNA/DNA Extraction Kit (CRIE, Moscow, Russia) according to the manufacturer’s instructions. Reverse transcription was carried out with a mixture of primers (Uni12, Uni12.4, and Uni13) [21] for type A influenza virus samples and the Uni11 primer for type B influenza virus samples using the reverse transcriptase M-MuLV-RH reagent kit (LLC BIOLABMIX, Novosibirsk, Russia). PCR amplification of cDNA was performed using the BioMaster LR HS-Taq PCR kit (2×) (LLC BIOLABMIX, Novosibirsk, Russia) according to the manufacturer’s instructions and previously described protocols with modifications [21,22]. Detailed protocols are available upon request. Deep sequencing of amplicons covering complete genomes was performed on an Illumina MiSeq using the MiSeq reagent kit v3 (Illumina, San Diego, CA, USA). The full-length genomes were assembled by the alignment of reads to known references with bwa-0.7.15 [23]. The obtained nucleotide sequences were deposited in the Global Initiative on Sharing All Influenza Data (GISAID) database. Phylogenetic analysis was performed using the maximum likelihood method based on the Hasegawa–Kishino–Yano model with 1000 bootstrap replications using MEGA 6.0 software (http://www.megasoftware.net/, accessed on 1 September 2023) [24]. For comparison, sequences of strains deposited in GISAID were used. Amino acid sequences were analyzed using FluSurver (http://flusurver.bii.a-star.edu.sg, accessed on 1 September 2023).

### 2.3. Virus Isolation and Antigenic Characterization

Seasonal influenza A and B viruses were isolated in MDCK cell culture from nasopharyngeal swabs or organ fragments (trachea, bronchi, or lungs) collected from patients diagnosed with influenza during the 2022–2023 flu season in Russia. Antigenic characterization of isolated seasonal influenza viruses was performed in a hemagglutination inhibition assay (HIA) with turkey red blood cells [20]. First, ferret antisera were treated with the receptor-destroying enzyme (RDE). For that, one volume of a post-infection ferret antiserum was mixed with three volumes of the RDE (II) reagent (Denka Seiken, Tokyo, Japan). The mixture was incubated at 37 °C for 16 h, followed by a 30 min incubation in a 56 °C water bath. Subsequently, six volumes of phosphate-buffered saline (PBS) were added to the mixture to obtain a 1:10 final serum dilution. Antisera were tested for the presence of nonspecific agglutinins. Then, antisera were used in the HIA. For that, serial two-fold dilutions of an antiserum were prepared in 25 µL of PBS. After that, a standardized dose (4 hemagglutination units) of a virus analyzed was added to prepared antiserum dilutions. Following the 30 min incubation at room temperature, 50 µL of a 0.5% suspension of turkey red blood cells (RBCs) were added to the reaction mixture. After a 45 min incubation at 4 °C, the results were registered. The highest antiserum dilution that inhibited hemagglutination was considered an HIA antiserum titer. For the characterization of A(H3N2) influenza viruses, 20 nM oseltamivir was added to the reaction buffer.

The characterization of influenza A(H1N1)pdm09 isolates was performed using the reference vaccine A(H1N1)pdm09 viruses (egg-propagated A/Victoria/2570/2019 and A/Guangdong-Maonan/SWL1536/2019; cell culture-propagated A/Wisconsin/588/2019) and the corresponding post-infection ferret antisera. For the characterization of A(H3N2) isolates, vaccine and reference strains A/Darwin/9/2021 (egg-grown), A/Stockholm/5/2021 (cell-grown), A/Cambodia/e0826360/2020 (egg-grown), and the corresponding ferret antisera were used. Analysis of influenza B/Victoria isolates was performed using cell culture- and egg-propagated variants of B/Washington/02/2019 and B/Austria/1359417/2021 vaccine viruses and the corresponding ferret antisera. All reference reagents (influenza viruses and ferret antisera) were kindly provided by the Worldwide Influenza Centre, WHO Collaborating Centre for Reference and Research on Influenza, The Francis Crick Institute, London, in 2021.

### 2.4. Phenotypic Analysis of Neuraminidase Inhibitors Susceptibility

Oseltamivir and zanamivir susceptibility of the isolated seasonal influenza viruses was tested using fluorescent neuraminidase inhibition assay (NIA) with a 2-(4-methylumbelliferyl)-a-D-N-acetylneuraminic acid (MUNANA) substrate [25].

## 3. Results

### 3.1. Detection of Influenza Cases in Russia during the 2022–2023 Flu Season

In this study, clinical samples from 780 laboratory-confirmed influenza A and B cases were analyzed. Country-wide monitoring was focused on the first influenza cases registered during the season, cases in vaccinated patients, and cases with fatal outcomes. As a result, the genetic material of 563 A(H1N1)pdm09 influenza viruses (72.2%) (including 156 cases with fatal outcomes), 22 A(H3N2) influenza viruses (2.8%) (no cases with fatal outcomes), and 195 influenza B viruses (25.0%) (including 22 cases with fatal outcomes) was identified by RT-PCR.

Of the 156 laboratory-confirmed A(H1N1)pdm09 fatal cases, 15.4% were registered in children 0–17 years old, 49.4% in adults aged 18 to 64, and 35.2% in adults aged 65 years old and over. Out of the 22 laboratory-confirmed fatal cases of B/Victoria, 45.4% were registered in children 0–17 years old, 36.4% in adults aged 18 to 64, and 18.2% in adults aged 65 years old and over. In total, 71% of fatal A(H1N1)pdm09 cases and 68% of fatal influenza B cases were associated with risk groups according to the WHO criteria (the risk group for influenza included children aged between 6 months and 5 years, adults aged more than 65 years old, and individuals with chronic medical conditions) (Table 1).

Genome sequencing was performed for a representative subset of specimens, including samples positive for different types and subtypes of influenza viruses from various regions of the country. All specimens obtained from fatal influenza cases with a sufficient amount of viral RNA were sequenced. In total, 215 influenza viruses were sequenced during the 2022–2023 flu season. These included 163 A(H1N1)pdm09 (including 93 cases with fatal outcomes), five A(H3N2), and 47 influenza B/Victoria viruses (including 20 cases with fatal outcomes).

### 3.2. Genetic and Virological Analysis of Influenza A(H1N1)pdm09 Viruses

The phylogenetic analysis of 160 influenza A(H1N1)pdm09 viruses showed that the 159 viruses analyzed in this study belonged to the clade 5a.2a (characteristic HA AASs K54Q, A186T (antigenic site Sb), Q189E (antigenic site Sb), E224A (RBS), R259K, K308R), and one virus belonged to the clade 5a.2a.1 (additional characteristic HA AASs P137S (antigenic site Ca), K142R (antigenic site Ca), D260E, T277A, E356D, I418V, N451H) [6].

**Figure 1:**
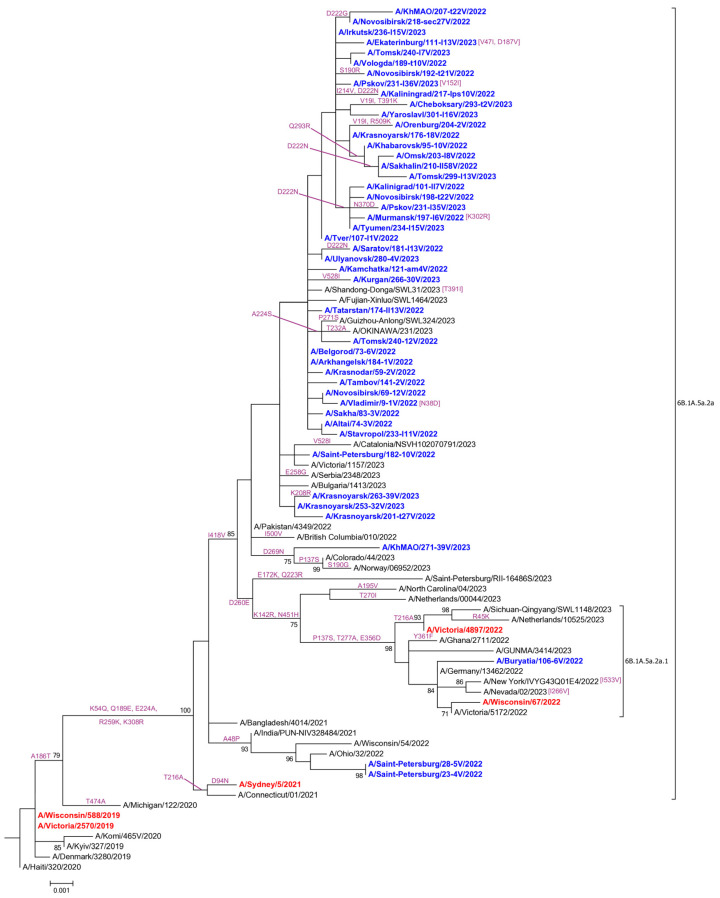
A maximum likelihood phylogenetic tree of the HA gene of influenza A(H1N1)pdm09 viruses analyzed in this study. Viruses isolated in Russia during the 2022–2023 epidemic season are indicated in blue. Vaccine strains are indicated in red. Amino acid substitutions in HA are in purple.

Of the 159 viruses from the 5a.2a clade, two had an additional AAS HA-A48P. The other 157 viruses from the 5a.2a clade had an additional AAS HA-I418V in the absence of A48P substitution. Of the 157 viruses bearing I418V AAS, one strain had HA-D269N AAS and was clustered with similar viruses that were registered sporadically in Europe and North America during the 2022–2023 season (Figure 1).

During the 2022–2023 influenza season, 69 influenza A(H1N1)pdm09 isolates were studied in hemagglutination inhibition assay (HIA) with post-infection ferret antisera. Out of the 69 viruses studied, 50 (72.5%) were isolated from fatal influenza cases (in 33 cases (47.8%), patients were not vaccinated, and vaccination status was unknown in 17 cases (24.7%)). The remaining 19 viruses (27.5%) were isolated from recovered patients, 16 (23.2%) of whom had been vaccinated against influenza prior to the 2022–2023 flu season (percentages out of the 69 isolates tested in HIA).

Of the 69 viruses studied in HIA, hemagglutinin genetic sequences were determined for 60 strains. The HA-based phylogenetic analysis showed that fifty-nine isolates of these belonged to the 5a.2a clade, while one isolate belonged to the 5a.2a.1 clade (A/Buryatia/106-6V/2022). Ferret antiserum raised against the cell culture-propagated A/Wisconsin/588/2019 strain (5a.2 clade) recognized 68 out of 69 studied viruses at titers 2–8-fold higher than the homologous titer. One virus (A/Novosibirsk/192-t21V/2022) bearing HA-S190R AAS reacted with this serum at a titer 2-fold lower than homologous. Similarly, ferret antiserum raised against the egg-propagated A/Victoria/2570/2019 strain (5a.2 clade) recognized 66 out of 69 viruses studied at titers equal to or 2-4-fold higher than homologous. Two isolates were recognized by this serum at titers 2-fold lower than homologous, and the mentioned above isolate A/Novosibirsk/192-t21V/2022 was recognized at a titer 8-fold lower than homologous. Additionally, 47 isolates out of 69 viruses studied with other antisera were tested in HIA with ferret antiserum raised against the A/Guangdong-Maonan/SWL1536/2019 strain from the heterologous 5a.1 clade from the 2020–2021 NH influenza vaccine. HIA results showed an 8–64-fold decrease in HI titers in the assay with these 47 isolates compared to the homologous serum HI titer (Tables S1 and S2).

Of the sixty-nine A(H1N1)pdm09 isolates tested in HIA, fourteen had HA-D222N AAS and two had HA-D222G AAS. All tested isolates bearing these substitutions were well-recognized by antisera raised against A/Victoria/2570/2019 and A/Wisconsin/588/2019 strains.

### 3.3. Genetic and Virological Analysis of Influenza A(H3N2) Viruses

During the 2022–2023 flu season, genome sequences of five influenza A(H3N2) viruses from several regions of Russia were determined. The phylogenetic analysis of HA gene sequences showed that these viruses belonged to one of the four new genetic subclades recently recognized within the clade 3C.2a1b.2a.2 (Figure S1).

One virus belonged to the 2a.1b subclade and had all AASs in HA (D53G, D104G, I140K, K276R, R299K), which were typical for this group.

One virus belonged to the 2a.3a.1 subclade and had all AASs in HA (E50K, D53N, N96S, I140K, I192F, I223V, N378S), which were typical for this subclade.

One virus analyzed belonged to the genetic subclade 2b characterized by the typical HA amino acid substitutions E50K, F79V, and I140K. Upon further evolution, this virus from the 2b subclade acquired several additional AASs in HA (F79I, T135A, and S262K).

Two sequenced A(H3N2) viruses belonged to the 2a.3b subclade and had all AASs in HA (D53N, N96S, I140M, I192F, and N378S), which were typical for this group.

During the 2022–2023 influenza season, we isolated only one influenza A(H3N2) virus (A/Khabarovsk/95-5V/2022) in MDCK cells. This strain was isolated from an unvaccinated person with mild influenza and belonged to the 2b genetic subclade. Analysis of this strain in HIA with turkey red blood cells in the presence of 20nM oseltamivir showed that the ferret antiserum raised against the egg-propagated 2022–2023 vaccine strain A/Darwin/9/2021 (subclade 2a) recognized this isolate at a titer that was one-fourth of the homologous. The serum raised against the cell-propagated A/Stockholm/5/2021 reference strain from the 2a subclade recognized this isolate at a titer that was half of the homologous. The antiserum raised against the egg-propagated 2021–2022 vaccine strain A/Cambodia/e0826360/2020 from the subclade 1a recognized the isolate at a titer that was 1/16th of the homologous (Table S3).

### 3.4. Genetic and Virological Analysis of Influenza B Viruses

Of the 195 clinical specimens positive for influenza B virus, 96 had a sufficient amount of genetic material, enabling the determination of influenza B virus lineage by RT-PCR. All 96 samples belonged to the B/Victoria lineage. None of the specimens tested were positive for B/Yamagata influenza viruses. Genome nucleotide sequences were determined for viruses originating from 47 influenza B cases, including 20 cases with fatal outcomes.

The phylogenetic analysis of the HA segment showed that all the viruses studied belonged to the V1A.3a.2 clade of the B/Victoria genetic lineage. All the viruses analyzed had three AASs in the HA characteristic for the V1A.3a.2 clade: A127T (in the 120 antigenic loop), P144L (in the 150 antigenic loop), and K203R. The characterized influenza B viruses were distributed between two genetic subgroups within the V1A.3a.2 clade. One of them had characteristic HA-E128K, A154E, and S208P AASs, while the other was defined by the D197E AAS with or without additional AASs R80G and E184K (Figure S2).

The mutations previously associated with increased virulence (according to FluSurver analysis and available published data [26]) were not detected in genomes of the B/Victoria viruses from fatal cases analyzed in this study.

During the 2022–2023 flu season, 23 influenza B/Victoria viruses were isolated in MDCK cells. They were tested in HIA with reference ferret antisera. Five viruses were isolated from fatal influenza cases in unvaccinated individuals. The remaining eighteen viruses were isolated from patients with non-fatal influenza, six of whom had been vaccinated against influenza and eleven had not been vaccinated, and in one case, the vaccination status was unknown. Hemagglutinin gene sequences were determined for seventeen out of twenty-three influenza B/Victoria viruses tested in HIA, including five viruses isolated from fatal influenza cases. All of them belonged to the V1A.3a.2 genetic clade. Nine isolates were attributed to the genetic subgroup characterized by HA-E128K, A154E, and S208P AASs in comparison with the vaccine strain B/Austria/1359417/2021. The remaining eight viruses belonged to the genetic subgroup bearing HA-D197E AAS in comparison with B/Austria/1359417/2021. Of these, six viruses had additional substitutions R80G and E184K. Ferret antisera raised against the cell culture and egg-propagated variants of the 2022–2023 NH B/Victoria vaccine strain B/Austria/1359417/2021 (clade V1A.3a.2) recognized all 23 tested isolates at titers equal or greater than homologous. Ferret antiserum raised against the egg-propagated 2021–2022 vaccine strain B/Washington/02/2019 from the genetic clade V1A.3 recognized all tested isolates at titers that were 8–16 times less than homologous. Eight B/Victoria isolates out of twenty-three tested viruses were recognized by ferret antiserum to the cell culture-propagated variant of the B/Washington/02/2019 strain at titers of no less than 1/4th of homologous (Tables S4 and S5).

### 3.5. Evaluation of HA-D222G/N Polymorphism in Influenza A(H1N1)pdm09 Viruses

During the 2022–2023 flu season, 163 influenza A(H1N1)pdm09 viruses were analyzed for the presence of HA-D222G and D222N polymorphisms using targeted next-generation sequencing (NGS). Of the 163 analyzed influenza A(H1N1)pdm09 viruses, 93 viruses were from influenza cases with fatal outcomes. Clinical material from cases with recovery included the upper respiratory tract (URT) samples (nasopharyngeal swabs), whereas clinical material from cases with fatal outcomes included only autopsy tissue samples from the lower respiratory tract (trachea, bronchi, or lung).

Of the 93 analyzed A(H1N1)pdm09 fatal cases, pneumonia was diagnosed in 47 cases (50.5%).

**Figure 2:**
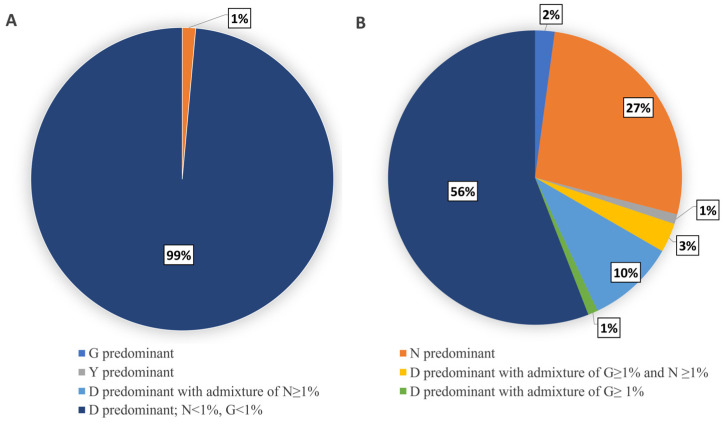
Pie charts representing the proportions of influenza A(H1N1)pdm09 virus samples with different intra-sample content of viral subpopulations bearing polymorphisms at the 222 codon. (**A**). Influenza cases with recovery (N = 70). (**B**). Fatal influenza cases (N = 90).

The analysis showed that in the majority of the 70 virus specimens originating from influenza cases with recovery (original nasopharyngeal swabs and MDCK isolates), wild type viruses with the HA-222D genotype were predominant. There was only one specimen originating from the upper respiratory tract of a recovered individual in which the virus variant with the 222N genotype prevailed. All of the remaining 69 tested specimens from influenza cases with recovery were characterized by overwhelming intra-sample predominance of the 222D genotype without significant (>1% of viral population in a sample) admixture of 222G or 222N variants (Figure 2A).

Among specimens originating from fatal influenza cases, virus subpopulations with polymorphisms at the 222 HA codon were registered more frequently. Of the 93 analyzed samples from fatal cases, only 52 were characterized by the intra-sample predominance of the wild type 222D genotype without a significant admixture (>1% of viral population) of 222N and 222G variants. In another 13 samples, the predominant wild type 222D variant coexisted with minority viral subpopulations bearing the 222N- or/and 222G-encoding genotype. They comprised 1–31.5% of the total viral population in a sample. In the remaining 28 samples from fatal cases, the predominant genotype differed from the wild type. In particular, 25 samples were characterized by the predominance of 222N variants in a sample. In two cases, 222G variants prevailed, while in one case, the dominance of the 222Y variant was identified (Figure 2B, Table S6).

**Figure 3:**
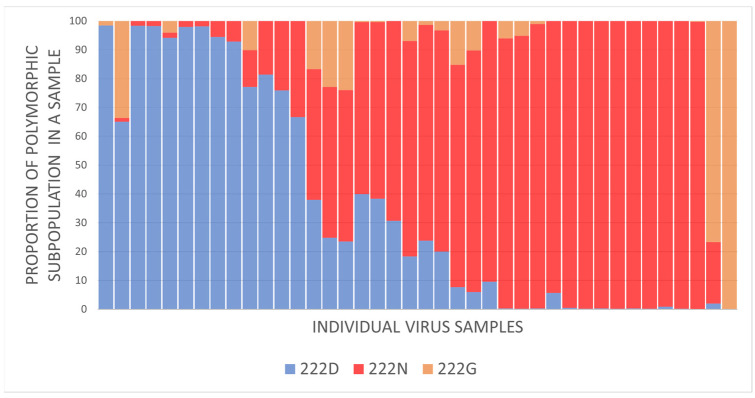
A normalized stacked bar chart representing profiles of the intra-sample genetic diversity of 40 individual influenza A(H1N1)pdm09 virus specimens originating from fatal influenza cases and containing at least 1% of HA-D222G and/or 1% of HA-D222N polymorphic variants.

In total, in 40 specimens from fatal influenza cases, the intra-sample content of virus variants with the 222N or 222G genotype exceeded 1% (Figure 3). Of these, nine samples had both polymorphic variants (222N and 222G), comprising at least 5% of the total intra-sample viral population each.

**Figure 4:**
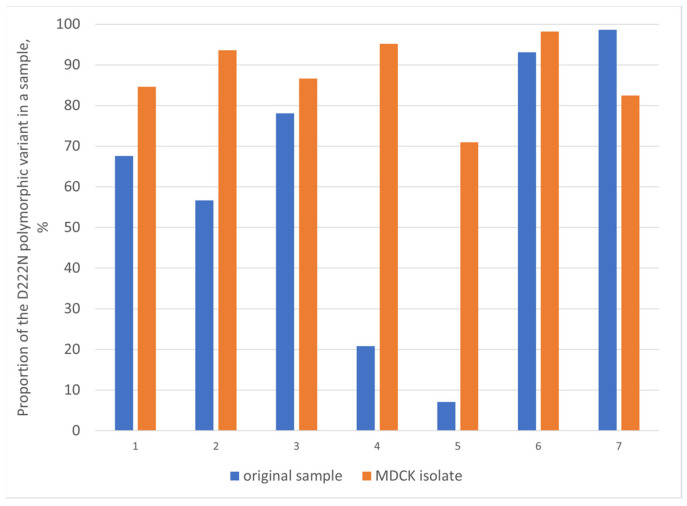
Comparison of the HA-D222N polymorphic virus variant content in the original clinical sample and the corresponding MDCK isolate for seven different influenza A(H1N1)pdm09 cases.

For seven influenza cases with HA-222 polymorphism, NGS sequencing was carried out both for the original material and the corresponding MDCK isolate. Based on these data, we were able to analyze the dynamic of changes in the content of different genetic subpopulations upon cultivation in an MDCK cell culture. In five cases of comparison, the D222N virus variant was predominant in the original clinical material and remained predominant in the corresponding MDCK isolate. In the other two cases, the wild type 222D genotype prevailed in the original sample with 7% and 21% admixture of 222N variants. The proportion of the 222N variant in the corresponding MDCK isolates increased 10 and 4.6 times for these cases, respectively (Figure 4).

Of the specimens with a dominating wild type 222D subpopulation and without significant (>1%) admixture of 222N and 222G variants, 26 specimens had NGS coverage of over 20,000 reads per 222 codons. This enabled the analysis of low-frequency 222 polymorphic variants comprising 0.1–1% of the total viral population in a sample. The results showed that all twenty-six analyzed specimens, including nineteen samples from fatal influenza cases and seven samples from cases with recovery, had a low-frequency admixture (0.2–0.8%) of 222N or/and 222G variants.

### 3.6. Drug Susceptibility

The genetic analysis of the NA segment of 192 influenza viruses (including 140 A(H1N1)pdm09, five A(H3N2), and 47 influenza B/Victoria strains) showed that the NA of 191 viruses did not have molecular markers of reduced susceptibility to neuraminidase inhibitors (according to the list of markers recommended by the WHO) [27]. One A(H1N)pdm09 strain A/Cheboksary/293-t2V/2023 (MDCK isolate) had AAS R152K in NA that was previously associated with reduced NA inhibition by oseltamivir, zanamivir, and laninamivir [27]. For this influenza case, sequencing of the original trachea tissue specimen was attempted, but the NA segment gene sequence was not determined successfully.

Polymerase acidic (PA) protein sequences of 136 influenza viruses were analyzed for the presence of AASs, which were previously associated with reduced susceptibility to baloxavir (according to the list of genetic markers recommended by the WHO) [28]. None of the viruses analyzed had markers associated with baloxavir resistance.

In addition to the genetic analysis of antiviral susceptibility, a subset of influenza viruses isolated in Russia during the 2022–2023 season were tested in fluorescence NIA. The objective was to determine their phenotypic susceptibility to neuraminidase inhibitors (NAIs) oseltamivir and zanamivir. In total, ninety influenza A(H1N1)pdm09, seventeen B/Victoria, and one A(H3N2) viruses were tested in NIA.

According to the WHO antiviral working group criteria [29], influenza viruses are considered to have reduced inhibition by NAIs in case their IC_50_ values exceed the median IC_50_ of viruses of the same subtype at least ten times for influenza A viruses or five times for influenza B viruses.

The median IC_50_ values of oseltamivir and zanamivir determined for influenza A(H1N1)pdm09 isolates were 0.22 and 0.32 nM, respectively. Of the ninety isolates tested, there was only one with oseltamivir IC_50_ exceeding the median value of the A(H1N1)pdm09 subtype by more than ten times (A/Cheboksary/293-t2V/2023 with AAS R152K in NA). The oseltamivir IC_50_ determined for this strain was 45 times higher than the subtype median. In zanamivir susceptibility analysis, the IC_50_ of the strain A/Cheboksary/293-t2V/2023 was 9–12 times greater than the A(H1N1)pdm09 subtype median (based on the results of three independent experiments). This lies on the border between normally inhibited viruses and viruses with reduced inhibition according to the WHO criteria. All the rest tested A(H1N1)pdm09 isolates were susceptible to zanamivir.

**Figure 5:**
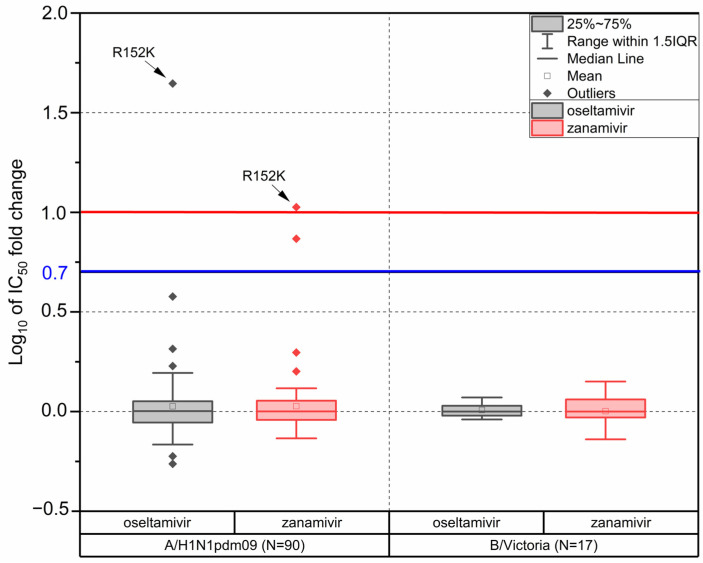
Box-and-whisker plot analysis of log-transformed IC_50_ fold changes of individual strains compared to the median IC_50_ determined for all viruses of the same subtype analyzed during the 2022–2023 influenza season (90 A(H1N1)pdm09 and 17 B/Victoria viruses). Red and blue horizontal lines represent threshold levels of log-transformed IC_50_ fold changes above which neuraminidase inhibition is considered to be reduced for influenza A and B viruses, respectively.

The median oseltamivir and zanamivir IC_50_ determined for the 17 tested influenza B/Victoria isolates were 17.31 and 3.77 nM, respectively. None of the viruses tested had oseltamivir or zanamivir IC_50_ values exceeding the subtype median more than five times (Figure 5, Table S7).

The oseltamivir and zanamivir IC_50_ values of the one tested A(H3N2) virus were 0.18 and 1.00 nM, respectively, which was within the typical range of normally inhibited influenza A(H3N2) viruses.

## 4. Discussion

### 4.1. Re-Emergence of a Widespread Circulation of Influenza A(H1N1pdm09) and B/Victoria Viruses in Russia in 2022–2023

The 2022–2023 epidemic season in Russia was characterized by the dominance of A(H1N1)pdm09 viruses. These viruses re-emerged in epidemic-scale circulation after two years of near absence since the beginning of the COVID-19 pandemic [8]. In this study, an analysis of a geographically representative subset of samples from influenza cases (including cases with fatal outcomes) showed that most of the viruses belonged to the A(H1N1)pdm09 subtype, representing a new antigenic clade 6B.1A.5a.2a. A smaller proportion of viruses belonged to influenza type B (B/Victoria lineage), representing the new clade V1A.3a.2. In addition, a few sporadic cases of A(H3N2) were detected and characterized.

The unprecedented fall in influenza virus circulation in 2020–2021 and, in particular, the near absence of A(H1N1)pdm09 in 2021–2022 in Russia may possibly be related to COVID-19 pandemic-related factors, such as viral interference and pandemic mitigation measures [3,30].

The observed dominance of A(H1N1)pdm09 viruses in Russia in 2022–2023 was different from the observed overall A(H3N2) dominance in the Northern Hemisphere. However, the predominant circulation of influenza A(H1N1)pdm09 viruses in Russia corresponded to the patterns of influenza circulation in central Asian countries [6].

In this country-wide monitoring, the proportion of A(H1N1)pdm09 cases (87.6%) was higher than influenza B cases (12.4%) among the laboratory-confirmed fatal influenza cases investigated in Russia during the 2022–2023 flu season. At the same time, the difference in the proportion of influenza A(H1N1)pdm09 and B viruses in the general circulation in Russia during the 2022–2023 season was less pronounced (56.4% and 34.3%, respectively), according to the previously reported sentinel surveillance data [8]. The results of the previously published studies, focused on the comparative analysis of disease severity and mortality of influenza caused by viruses of different types and subtypes, varied depending on the geographical region and methods used in the analysis. Increased hospitalization and mortality for influenza A(H1N1)pdm09, compared to A(H3N2) and influenza B, was shown in a previously reported study [31]. In another reported study, higher mortality on average was associated with influenza A(H3N2) virus [32]. In addition, other studies showed the association of influenza A(H1N1)pdm09 with increased severity of hospitalized cases [33] and a much higher risk of pneumonia in hospitalized cases [32]. It is important to note that influenza B viruses may cause relatively more severe pediatric cases compared to influenza A, as was previously reported [34,35].

### 4.2. Genetic and Antigenic Characterization of Influenza Viruses

#### 4.2.1. Influenza A(H1N1)pdm09 Viruses

A(H1N1)pdm09 influenza viruses from the 5a.2 clade have begun to circulate around the world and in Russia during the 2019–2020 flu season. They were characterized by the presence of the HA-N156K AAS and significant antigenic difference from the 5a.1 clade, as well as other previously circulated A(H1N1)pdm09 viruses [36]. The viruses analyzed in this study over the 2022–2023 season belonged to the descendent clades 5a.2a and 5a.2a.1 [6], with the 5a.2a clade being vastly predominant in Russia. At the same time, A(H1N1)pdm09 viruses from both clades were represented equally in other parts of Europe. In the USA, on the contrary, viruses of the 5a.2a.1 clade dominated among A(H1N1)pdm09 strains [37].

The antigenic characterization of A(H1N1)pdm09 viruses isolated in this study showed that the vast majority of 5a.2a clade isolates and the 5a.2a.1 isolate were antigenically similar to the 2022–2023 vaccine strains A/Victoria/2570/2019 and A/Wisconsin/588/2019 from the 5a.2 clade. This indicates that the AASs accumulated in the clades currently circulating did not result in a significant modification of antigenic properties compared to the 5a.2 genetic group, which could be distinguished by ferret antisera. We identified only one antigenically different isolate, which had HA-S190R AAS in the Sb antigenic site. At the same time, all the isolates tested differed dramatically from the 2020–2021 vaccine strain A/Guangdong-Maonan/SWL1536/2019 from the 5a.1 clade which was prevalent before the COVID-19 pandemic.

The antigenic similarity of A(H1N1)pdm09 viruses from the 5a.2a and 5a.2a.1 clades with the A/Victoria/2570/2019 strain was also shown in HIA with ferret antisera for the viruses of the 2022–2023 epidemic season in China [38], European countries [5], and the USA [39]. Nevertheless, WHO experts recommended replacing the A(H1N1)pdm09 vaccine component A/Victoria/2570/2019 (5a.2) with the A/Victoria/4897/2022 (5a.2a.1 clade) strain for the 2023–2024 NH flu season [6].

#### 4.2.2. Influenza A(H3N2) Viruses

The circulation of influenza A(H3N2) viruses in Russia during the 2022–2023 season was sporadic and accounted only for 0.6% of all registered positive influenza cases [8]. Of the few A(H3N2) viruses analyzed in our study, there were representatives of the new 2b, 2a.1b, 2a.3a.1, and 2a.3b subclades [6].

The global influenza surveillance of A(H3N2) viruses coordinated by the WHO showed that during the 2022–2023 flu season, multiple genetic subgroups of the predominant 3C.2a1b.2a.2 clade cocirculated in different parts of the world, with the subclades 2b, 2a.1b, and 2a.3a.1 being the most common. Various levels of antigenic similarity were observed between these subclades. Considering all the factors, the WHO recommended that A/Darwin/09/2021-like viruses be kept as an A(H3N2) component of the flu vaccine for the 2023–2024 NH season [6].

#### 4.2.3. Influenza B Viruses

All influenza B/Victoria viruses characterized in our study in Russia in 2022–2023 belonged to the new genetic group V1A.3a.2. The viruses further diversified into two genetic subgroups inside the clade V1A.3a.2. One of them had HA-D197E AAS, while the other had HA-E128K, A154E, and S208P AASs. The viruses from both subgroups were antigenically similar to the 2022–2023 vaccine strain B/Austria/1359417/2021 and differed from the 2021–2022 vaccine strain B/Washington/02/2019.

Influenza B/Victoria viruses from the clade V1A.3a.2 started being registered in the world toward the end of 2020. They were first detected in Russia at the end of the 2020–2021 flu season. Nevertheless, during the 2021–2022 flu season, influenza B viruses accounted for only 3.2% of all influenza cases [40]; they were vastly outnumbered in Russia by A(H3N2) viruses. Thus, influenza B viruses of the clade V1A.3a.2 representing new antigenic properties compared to the B/Washington/02/2019-like strains became widespread in Russia for the first time during the 2022–2023 season.

The vast majority of influenza B/Victoria viruses detected in the other parts of the world during the 2022–2023 flu season were also attributed to the genetic clade V1A.3a.2. These viruses continued to diversify [7].

The data published by the influenza surveillance centers in Europe, the USA, and China also indicate that the majority of currently circulating B/Victoria viruses are well recognized by antisera raised against the B/Austria/1359417/2021 strain [38,39,41]. Therefore, WHO experts recommended keeping the strain B/Austria/1359417/2021 as a B/Victoria component of the flu vaccine for the upcoming 2023–2024 influenza season [6].

### 4.3. Drug Susceptibility of Influenza Viruses

The genetic analysis of all the viruses studied showed that only one A(H1N1)pdm09 virus had the marker of reduced susceptibility to neuraminidase inhibitors [27]. The identified strain A/Cheboksary/293-t2V/2023 with the NA-R152K AAS showed reduced inhibition by oseltamivir and zanamivir with 45- and 12-fold changes in IC_50_, respectively. The level of reduction determined in oseltamivir and zanamivir inhibition was comparable to earlier published data for the A(H1N1)pdm09 virus with NA-R152K AAS [42]. All the other assessed viruses did not have genetic markers of drug resistance to NAIs and baloxavir [27,28] and were normally inhibited by oseltamivir and zanamivir.

Globally, viruses with reduced susceptibility to NAIs and PA inhibitors remain rare [6,7].

### 4.4. Evaluation of HA-D222G/N Polymorphism in Influenza A(H1N1)pdm09 Viruses

Previous studies showed that a substantial proportion of influenza A(H1N1)pdm09 viruses originating from fatal cases registered in Russia over several past seasons had a HA-D222G/N polymorphism, which was previously associated with disease severity. These studies included the 2015–2016 [13] and 2017–2019 [10,11,12] flu seasons, in which the A(H1N1)pdm09 virus was either dominant or widespread in circulation. The D222G/N substitutions in RBS were previously associated with lung infection complications, such as viral pneumonia. This is due to the increased affinity of HA with these substitutions to alpha 2,3-linked sialic acid receptors that are found primarily in the lungs [43,44,45]. In our study, we investigated the presence of D222G/N substitutions in the HA of the newly emerged dominant clade of influenza A(H1N1)pdm09 viruses (5a.2a), which had a subset of clade-specific amino acid substitutions in RBS.

#### 4.4.1. Detection of HA-D222G/N Polymorphism in A(H1N1)pdm09 Viruses from Fatal Cases

The targeted NGS approach showed that viral subpopulations with HA-D222G or D222N substitutions were predominant in 27 A(H1N1)pdm09 virus specimens originating from fatal influenza cases (29% of all A(H1N1)pdm09 fatal cases assessed for the intra-sample genetic diversity during the 2022–2023 season in Russia). In most of these cases (93%), the predominance of the D222N virus variants was identified.

Further analysis of NGS data showed that 14% of the fatal influenza A(H1N1)pdm09 cases were characterized by the predominance of the wild type 222D virus subpopulations in a sample with a 1–31.5% admixture of HA-D222G or/and D222N virus variants.

The comparable frequency of influenza A(H1N1)pdm09 viruses with D222G/N substitutions among influenza cases with fatal outcomes were registered in Russia during the 2018–2019 flu season. Particularly, D222G or D222N polymorphic virus variants prevailed in 32% of virus samples originating from fatal cases. In 25% of fatal cases, the predominating wild type virus variant coexisted with an admixture of D222G or/and D222N subpopulations comprising at least 1% of the total viral population [10]. In contrast to the 2022–2023 flu season, D222G polymorphic virus variants were registered more frequently than D222N variants during the 2018–2019 flu season in Russia. A similar high occurrence and predominance of viruses with the D222G substitution in severe influenza cases were also reported in Germany during the 2010–2011 flu season [46]. The observed inter-seasonal difference may indicate that the preferred accumulation of D222G or D222N substitutions may depend on the genetic background of influenza A(H1N1)pdm09 viruses circulating during a particular flu season.

Of the virus samples with the D222G/N substitutions registered during the 2022–2023 season, a substantial proportion contained viruses with both D222G and D222N variants (with a 5% or more proportion in the samples). Previous studies also reported the co-occurrence of D222G and D222N variants in A(H1N1)pdm09 virus samples from severe cases [10,11,12,46,47]. Such polymorphic virus populations could possibly lead to a more severe course of disease due to faster virus adaptation [48].

It is significant that the polymorphism in the HA of influenza A(H1N1)pdm09 viruses is very restricted to either D222N or D222G variants. Viruses with the predominant subpopulation bearing other substitutions, such as D222Y/E/A/V, are very rarely observed in severe cases. However, their association with the severity of the disease has not been shown and, as previously reviewed, their effect on virus properties has not been determined [9,10].

#### 4.4.2. Limited Detection of HA-D222G/N Substitutions in Influenza A(H1N1)pdm09 Viruses in Broad Circulation

According to the GISAID data (accessed on 08.10.2023), the HA-D222G and D222N substitutions in influenza A(H1N1)pdm09 viruses occurred in approximately 0.1% and 0.6% of all A(H1N1)pdm09 HA sequences globally deposited during the 2022–2023 season, respectively. Other substitution variants were rarely detected, with only single cases of D222A, D222E, and D222Y reported in GISAID.

The analysis of the virus samples with a high sequencing coverage (over 20,000 reads per codon) showed that in all specimens with an overwhelming predominance of the wild type D222 variant (including samples from cases with recovery), an admixture of D222G and/or D222N variants at a proportion of 0.2–0.8% was also present. This observation is similar to the previously reported studies [10], indicating the ubiquitous presence of the low initial concentration of mutated virus variants. In the case of favorable selection conditions, the proportion of viruses with substitutions may increase.

The near absence of the detection of virus samples with D222G/N substitutions (with a proportion of over 1%) in the broad virus circulation observed in this and other studies [11,12,46,48,49], as well as the increased affinity of the mutant viruses to receptors of the lower respiratory tract (LRT), support the hypothesis that in most cases, the accumulation of viruses with the D222G/N substitutions results from a selection from the polymorphic influenza virus population in the lower respiratory tract. Mutations may be absent or non-dominant at the time of infection [10,50].

In our study, we showed that polymorphic A(H1N1)pdm09 viral populations with HA-D222G and D222N substitutions were primarily detected in the viruses from the LRT tissue samples from influenza cases with fatal outcomes. Further analysis of the D222G/N polymorphism in URT (nasopharyngeal swabs) and LRT (sputum) samples obtained from patients with pneumonia at different stages of disease progression is needed for a better understanding of viral population dynamics over the course of the disease.

Despite the currently observed limited representation of D222G/N polymorphism in influenza A(H1N1)pdm09 viruses in broad circulation, it is important to monitor the circulation of viruses with these substitutions and detect epidemiologically significant shifts in circulation and changes in virus properties, such as transmissibility and pathogenicity.

The ability of the A(H1N1)pdm09 virus to rapidly accumulate HA-D222G/N substitutions and thus adapt to human LRT is an important and insufficiently understood pathogenicity factor. It needs to be further investigated and taken into consideration in the aims of the possible optimization of prevention and treatment strategies, particularly for patients from the risk groups.

The lack of a significant effect of HA-D222G/N substitutions on the antigenic properties of the A(H1N1)pdm09 virus has been shown in this work, our previous publications [11,12], and other reported studies [51]. This indicates that vaccination remains an effective protection means against virus variants with HA-D222G/N substitutions.

## 5. Conclusions

A re-emergence of a large-scale circulation of influenza A(H1N1)pdm09 viruses was observed in Russia during the 2022–2023 flu season. It was characterized by the dominance of the new 6B.1A.5a.2a clade viruses, which were antigenically distinct from the 5a.1 A(H1N1)pdm09 viruses present in circulation prior to the start of the COVID-19 pandemic. Influenza B viruses, which also reentered wide circulation in 2022–2023, belonged to the new V1A.3a.2 clade.

A substantial number of A(H1N1)pdm09 virus samples (29%) from fatal influenza cases were predominated by subpopulations bearing HA-D222N or D222G amino acid substitutions, which may represent an important pathogenicity factor associated with severe influenza cases.

The return of influenza to epidemic-level circulation and the high rate of evolution of seasonal influenza viruses with the emergence of new antigenic clades and pathogenicity factors highlights the importance of monitoring seasonal influenza, including severe cases, with the aim of developing and optimizing prevention and treatment measures.

## Figures and Tables

**Table 1:** Data on the studied laboratory-confirmed influenza cases.

Data on Cases	Total	A(H1N1)pdm09	A(H3N2)	Influenza B
Number of laboratory-confirmed cases/proportion	780	563 (72.2%)	22 (2.8%)	195 (25%)
Number of cases with fatal outcomes/proportion	178	156 (87.6%)	0	22 (12.4%)
	Age group, years old			
Proportion of patients in different age groups among cases with fatal outcomes	0–17	15.4%	-	45.4%
	18–64	49.4%	-	36.4%
	65 and over	35.2%	-	18.2%
Proportion of patients in risk groups for influenza among cases with fatal outcomes		71%	-	68%

## Data Availability

The data presented in this study are available in the article and supplementary materials.

## Supplementary Materials

The following supporting information can be downloaded at https://www.mdpi.com/article/10.3390/pathogens13010001/s1, Figure S1: A(H3N2) HA phylogenetic tree; Figure S2: B/Victoria HA phylogenetic tree; Table S1: HIA assay results for individual A(H1N1)pdm09 isolates; Table S2: Summary table of the A(H1N1)pdm09 HIA results; Table S3: HIA assay results for the A(H3N2) isolate; Table S4: HIA assay results for individual B/Victoria isolates; Table S5: Summary table of the B/Victoria HIA results; Table S6: Analysis of the HA-222D/G/N polymorphism in the A(H1N1)pdm09 virus samples; Table S7: IC_50_ of NAIs determined for isolated seasonal influenza viruses.

## References

[1] Uyeki T.M. (2020). High-risk Groups for Influenza Complications. JAMA.

[2] Adlhoch C., Mook P., Lamb F., Ferland L., Melidou A., Amato-Gauci A.J., Pebody R., Network T.E.I.S. (2020). Decreased Influenza Activity During the COVID-19 Pandemic—United States, Australia, Chile, and South Africa, 2020. MMWR Morb. Mortal. Wkly. Rep..

[3] Adlhoch C., Mook P., Lamb F., Ferland L., Melidou A., Amato-Gauci A.J., Pebody R. (2021). European Influenza Surveillance Network.Very little influenza in the WHO European Region during the 2020/21 season, weeks 40 2020 to 8 2021. Euro Surveill..

[4] Bolton M.J., Ort J.T., McBride R., Swanson N.J., Wilson J., Awofolaju M., Furey C., Greenplate A.R., Drapeau E.M., Pekosz A. (2022). Antigenic and virological properties of an H3N2 variant that continues to dominate the 2021–22 Northern Hemisphere influenza season. Cell Rep..

[5] (2023). Influenza Virus Characterization: Summary Report, Europe, February 2023.

[6] World Health Organization (2023). Recommended Composition of Influenza Virus Vaccines for Use in the 2023–2024 Northern Hemisphere Influenza Season—Composition Recommandée des Vaccins Antigrippaux pour la Saison Grippale 2023–2024 dans L’hémisphère Nord. Wkly Epidemiol. Rec..

[7] (2023). World Health Organization. Recommended Composition of Influenza Virus Vaccines for Use in the 2024 Southern Hemisphere Influenza Season—Composition Recommandée des Vaccins Antigrippaux pour la Saison Grippale 2024 dans L’hémisphère Sud Wkly Epidemiol. Rec..

[8] Sominina A., Danilenko D., Komissarov A.B.B., Pisareva M., Fadeev A.V., Konovalova N., Eropkin M., Petrova P., Zheltukhina A., Musaeva T.D. (2023). Assessing the Intense Influenza A(H1N1)pdm09 Epidemic and Vaccine Effectiveness in the Post-COVID Season in the Russian Federation. Viruses.

[9] Goka E.A., Vallely P.J., Mutton K.J., Klapper P.E. (2014). Mutations associated with severity of the pandemic influenza A(H1N1)pdm09 in humans: A systematic review and meta-analysis of epidemiological evidence. Arch. Virol..

[10] Danilenko A.V., Kolosova N.P., Shvalov A.N., Ilyicheva T.N., Svyatchenko S.V., Durymanov A.G., Bulanovich J.A., Goncharova N.I., Susloparov I.M., Marchenko V.Y. (2021). Evaluation of HA-D222G/N polymorphism using targeted NGS analysis in A(H1N1)pdm09 influenza virus in Russia in 2018–2019. PLoS ONE.

[11] Kolosova N.P., Ilyicheva T.N., Danilenko A.V., Svyatchenko S.V., Goncharova N.I., Bulanovich J.A., Torzhkova P.Y., Durymanov A.G., Gudymo A.S., Shvalov A.N. (2020). Severe cases of seasonal influenza and detection of seasonal A(H1N2) in Russia in 2018–2019. Arch. Virol..

[12] Kolosova N.P., Ilyicheva T.N., Danilenko A.V., Bulanovich J.A., Svyatchenko S.V., Durymanov A.G., Goncharova N.I., Gudymo A.S., Shvalov A.N., Susloparov I.M. (2019). Severe cases of seasonal influenza in Russia in 2017-2018. PLoS ONE.

[13] Ilyicheva T., Durymanov A., Susloparov I., Kolosova N., Goncharova N., Svyatchenko S., Petrova O., Bondar A., Mikheev V., Ryzhikov A. (2016). Fatal Cases of Seasonal Influenza in Russia in 2015–2016. PLoS ONE.

[14] Reid A.H., Janczewski T.A., Lourens R.M., Elliot A.J., Daniels R.S., Berry C.L., Oxford J.S., Taubenberger J.K. (2003). 1918 influenza pandemic caused by highly conserved viruses with two receptor-binding variants. Emerg. Infect. Dis..

[15] World Health Organization (2010). Preliminary Review of D222G Amino Acid Substitution in the Haemagglutinin of Pandemic Influenza A (H1N1) 2009 Viruses. [Internet]. Vol. 85, Weekly Epidemiological Record = Relevé Épidémiologique Hebdomadaire. https://apps.who.int/iris/handle/10665/241505.

[16] Zheng B., Chan K.-H., Zhang A.J.X., Zhou J., Chan C.C.S., Poon V.K.M., Zhang K., Leung V.H.C., Jin D.-Y., Woo P.C.Y. (2010). D225G mutation in hemagglutinin of pandemic influenza H1N1 (2009) virus enhances virulence in mice. Exp. Biol. Med..

[17] Xu L., Bao L., Lv Q., Deng W., Ma Y., Li F., Zhan L., Zhu H., Ma C., Qin C. (2010). A single-amino-acid substitution in the HA protein changes the replication and pathogenicity of the 2009 pandemic A (H1N1) influenza viruses in vitro and in vivo. Virol. J..

[18] Chan K.-H., Zhang A.J.X., To K.K.W., Chan C.C.S., Poon V.K.M., Guo K., Ng F., Zhang Q.-W., Leung V.H.C., Cheung A.N.Y. (2010). Wild type and mutant 2009 pandemic influenza a (H1N1) viruses cause more severe disease and higher mortality in pregnant balb/c mice. PLoS ONE.

[19] Belser J.A., Jayaraman A., Raman R., Pappas C., Zeng H., Cox N.J., Katz J.M., Sasisekharan R., Tumpey T.M. (2011). Effect of D222G mutation in the hemagglutinin protein on receptor binding, pathogenesis and transmissibility of the 2009 pandemic H1N1 influenza virus. PLoS ONE.

[20] World Health Organization (2011). Manual for the Laboratory Diagnosis and Virological Surveillance of Influenza.

[21] St George K. (2012). Diagnosis of Influenza Virus. Influenza Virus Methods and Protocols.

[22] Deng Y.-M., Spirason N., Iannello P., Jelley L., Lau H., Barr I.G. (2015). A simplified Sanger sequencing method for full genome sequencing of multiple subtypes of human influenza A viruses. J. Clin. Virol..

[23] Li H. (2013). Aligning sequence reads, clone sequences and assembly contigs with BWA-MEM. arXiv.

[24] Tamura K., Stecher G., Peterson D., Filipski A., Kumar S. (2013). MEGA6: Molecular Evolutionary Genetics Analysis Version 6.0. Mol. Biol. Evol..

[25] Leang S.-K., Hurt A.C. (2017). Fluorescence-based Neuraminidase Inhibition Assay to Assess the Susceptibility of Influenza Viruses to The Neuraminidase Inhibitor Class of Antivirals. J. Vis. Exp..

[26] Zhou L., Feng Z., Liu J., Chen Y., Yang L., Liu S., Li X., Gao R., Zhu W., Wang D. (2020). A single N342D substitution in Influenza B Virus NA protein determines viral pathogenicity in mice. Emerg. Microbes Infect..

[27] Summary of Neuraminidase (NA) Amino Acid Substitutions Associated with Reduced Inhibition by Neuraminidase Inhibitors (NAIs). https://www.who.int/publications/m/item/summary-of-neuraminidase-(na)-amino-acid-substitutions-associated-with-reduced-inhibition-by-neuraminidase-inhibitors-(nais).

[28] Summary of Polymerase Acidic (PA) Protein Amino Acid Substitutions Analysed for Their Effects On Baloxavir Susceptibility. https://www.who.int/publications/m/item/summary-of-polymerase-acidic-(pa)-protein-amino-acid-substitutions-analysed-for-their-effects-on-baloxavir-susceptibility.

[29] (2012). Meetings of the WHO working group on surveillance of influenza antiviral susceptibility—Geneva, November 2011 and June 2012. Wkly. Epidemiol. Rec..

[30] Jones R.P., Ponomarenko A. (2022). Roles for Pathogen Interference in Influenza Vaccination, with Implications to Vaccine Effectiveness (VE) and Attribution of Influenza Deaths. Infect. Dis. Rep..

[31] Lytras T., Andreopoulou A., Gkolfinopoulou K., Mouratidou E., Tsiodras S. (2019). Association between type-specific influenza circulation and incidence of severe laboratory-confirmed cases; which subtype is the most virulent?. Clin. Microbiol. Infect..

[32] Goldstein E. (2022). Mortality Associated with Different Influenza Subtypes in France between 2015–2019. medRxiv.

[33] Sumner K.M., Masalovich S., O’Halloran A., Holstein R., Reingold A., Kirley P.D., Alden N.B., Herlihy R.K., Meek J., Yousey-Hindes K. (2023). Severity of influenza-associated hospitalisations by influenza virus type and subtype in the USA, 2010–2019: A repeated cross-sectional study. Lancet Microbe.

[34] Pediatric Flu Deaths during 2019–2020 Reach New High. https://www.cdc.gov/flu/spotlights/2020-2021/pediatric-flu-deaths-reach-new-high.htm.

[35] Özkaya P.Y., Turanli E.E., Metïn H., Uysal A.A., Çïçek C., Karapinar B. (2021). Severe influenza virus infection in children admitted to the PICU: Comparison of influenza A and influenza B virus infection. J. Med. Virol..

[36] World Health Organization (2020). Recommended Composition of Influenza Virus Vaccines for Use in the 2021 Southern Hemisphere Influenza Season—Composition Recommandée des Vaccins Antigrippaux pour la Saison GRIPPALE 2021 dans l’hémisphère Sud. Wkly. Epidemiol. Rec..

[37] Worldwide Influenza Centre, WHO CC for Reference and Research on Influenza, The Francis Crick Institute Report Prepared for the WHO Annual Consultation on the Composition of Influenza Vaccines for the Northern Hemisphere 2023–2024. https://www.crick.ac.uk/sites/default/files/2023-03/F2023-VCM-seasonal_web.pdf.

[38] Chinese Weekly Influenza Surveillance Report. September 25 to October 1, 2023 (Week 39). https://ivdc.chinacdc.cn/cnic/en/Surveillance/WeeklyReport/202310/P020231007282742073293.pdf.

[39] Influenza Activity in the United States during the 2022–23 Season and Composition of the 2023–24 Influenza Vaccine. https://www.cdc.gov/flu/spotlights/2023-2024/22-23-summary-technical-report.htm.

[40] Sominina A., Danilenko D., Komissarov A., Karpova L., Pisareva M., Fadeev A., Konovalova N., Eropkin M., Stolyarov K., Shtro A. (2022). Resurgence of Influenza Circulation in the Russian Federation during the Delta and Omicron COVID-19 Era. Viruses.

[41] (2023). Influenza Virus Characterization: Summary Report, Europe, August 2023.

[42] Govorkova E.A., Takashita E., Daniels R.S., Fujisaki S., Presser L.D., Patel M.C., Huang W., Lackenby A., Nguyen H.T., Pereyaslov D. (2022). Global update on the susceptibilities of human influenza viruses to neuraminidase inhibitors and the cap-dependent endonuclease inhibitor baloxavir, 2018–2020. Antivir. Res..

[43] L’vov D.K., Burtseva E.I., Prilipov A.G., Bogdanova V.S., Shchelkanov M.I., Bovin N.V., Samokhvalov E.I., Fediakina I.T., Deriabin P.G., Kolobukhina L.V. (2010). A possible association of fatal pneumonia with mutations of pandemic influenza A/H1N1 sw1 virus in the receptor-binding site of the HA1 subunit. Vopr. Virusol..

[44] Chutinimitkul S., Herfst S., Steel J., Lowen A.C., Ye J., van Riel D., Schrauwen E.J.A., Bestebroer T.M., Koel B., Burke D.F. (2010). Virulence-associated substitution D222G in the hemagglutinin of 2009 pandemic influenza A(H1N1) virus affects receptor binding. J. Virol..

[45] Krasnoslobodtsev K.G., Lvov D.K., Alkhovsky S.V., Burtseva E.I., Fedyakina I.T., Kolobukhina L.V., Kirillova E.S., Trushakova S.V., Oskerko T.A., Shchelkanov M.Y. (2016). Amino acid polymorphism at residue 222 of the receptor-binding site of the hemagglutinin of the pandemic influenza A(H1N1)pdm09 from patients 166 with lethal virus pneumonia in 2012-2014. Probl. Virol..

[46] Wedde M., Wählisch S., Wolff T., Schweiger B. (2013). Predominance of HA-222D/G Polymorphism in influenza A(H1N1)pdm09 viruses associated with fatal and severe outcomes recently circulating in germany. PLoS ONE.

[47] Piralla A., Rovida F., Girello A., Premoli M., Mojoli F., Belliato M., Braschi A., Iotti G., Pariani E., Bubba L. (2017). Frequency of respiratory virus infections and next-generation analysis of influenza A/H1N1pdm09 dynamics in the lower respiratory tract of patients admitted to the ICU. PLoS ONE.

[48] Selleri M., Piralla A., Rozera G., Giombini E., Bartolini B., Abbate I., Campanini G., Rovida F., Dossena L., Capobianchi M. (2013). Detection of haemagglutinin D222 polymorphisms in influenza A(H1N1)pdm09-infected patients by ultra-deep pyrosequencing. Clin. Microbiol. Infect..

[49] Stevens J., Blixt O., Glaser L., Taubenberger J.K., Palese P., Paulson J.C., Wilson I.A. (2006). Glycan Microarray Analysis of the Hemagglutinins from Modern and Pandemic Influenza Viruses Reveals Different Receptor Specificities. J. Mol. Biol..

[50] Takayama I., Nguyen B.G., Dao C.X., Pham T.T., Dang T.Q., Truong P.T., Van Do T., Pham T.T.P., Fujisaki S., Odagiri T. (2021). Next-Generation Sequencing Analysis of the Within-Host Genetic Diversity of Influenza A(H1N1)pdm09 Viruses in the Upper and Lower Respiratory Tracts of Patients with Severe Influenza. mSphere.

[51] Abed Y., Pizzorno A., Hamelin M.-E., Leung A., Joubert P., Couture C., Kobasa D., Boivin G. (2011). The 2009 Pandemic H1N1 D222G Hemagglutinin Mutation Alters Receptor Specificity and Increases Virulence in Mice but Not in Ferrets. J. Infect. Dis..

