# Supplementary material for: An Investigation of Severe Influenza Cases in Russia during the 2022–2023 Epidemic Season and an Analysis of HA-D222G/N Polymorphism in Newly Emerged and Dominant Clade 6B.1A.5a.2a A(H1N1)pdm09 Viruses
Source: Pathogens. 2023 Dec 19;13(1):1. doi: 10.3390/pathogens13010001 (PMC10819184; doi:10.3390/pathogens13010001)
Supplement: Supplementary file 1 [file pathogens-13-00001-s001.zip › Supplementary Figures S1,S2.pdf]

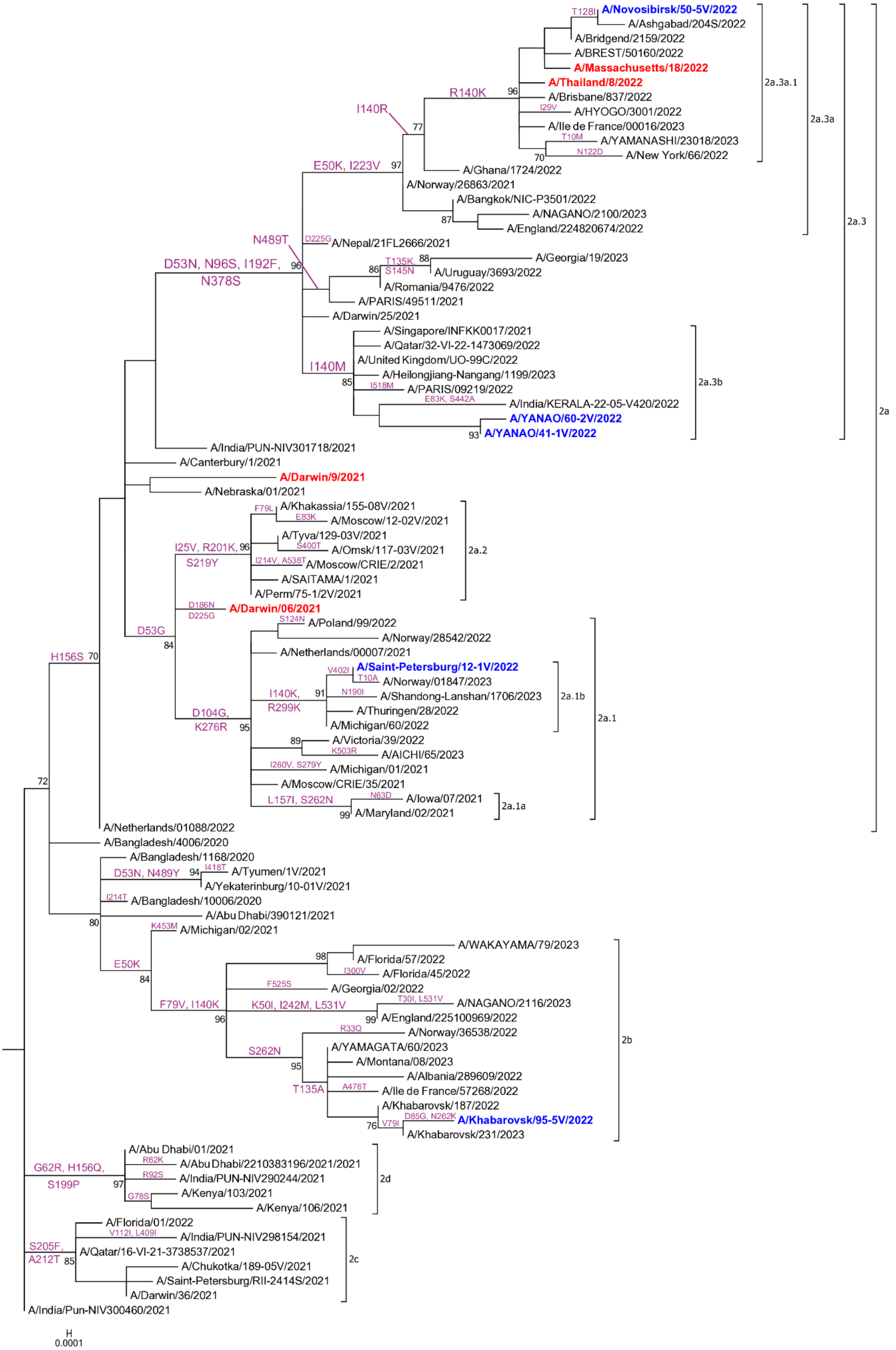

Figure S1. A(H3N2) HA phylogenetic tree. A maximum likelihood phylogenetic tree of the HA gene of influenza A(H3N2) viruses analyzed in this study. Viruses isolated in Russia during the 2022–2023 epidemic season are indicated in blue. Vaccine strains are indicated in red. Amino acid substitutions in HA are in purple.

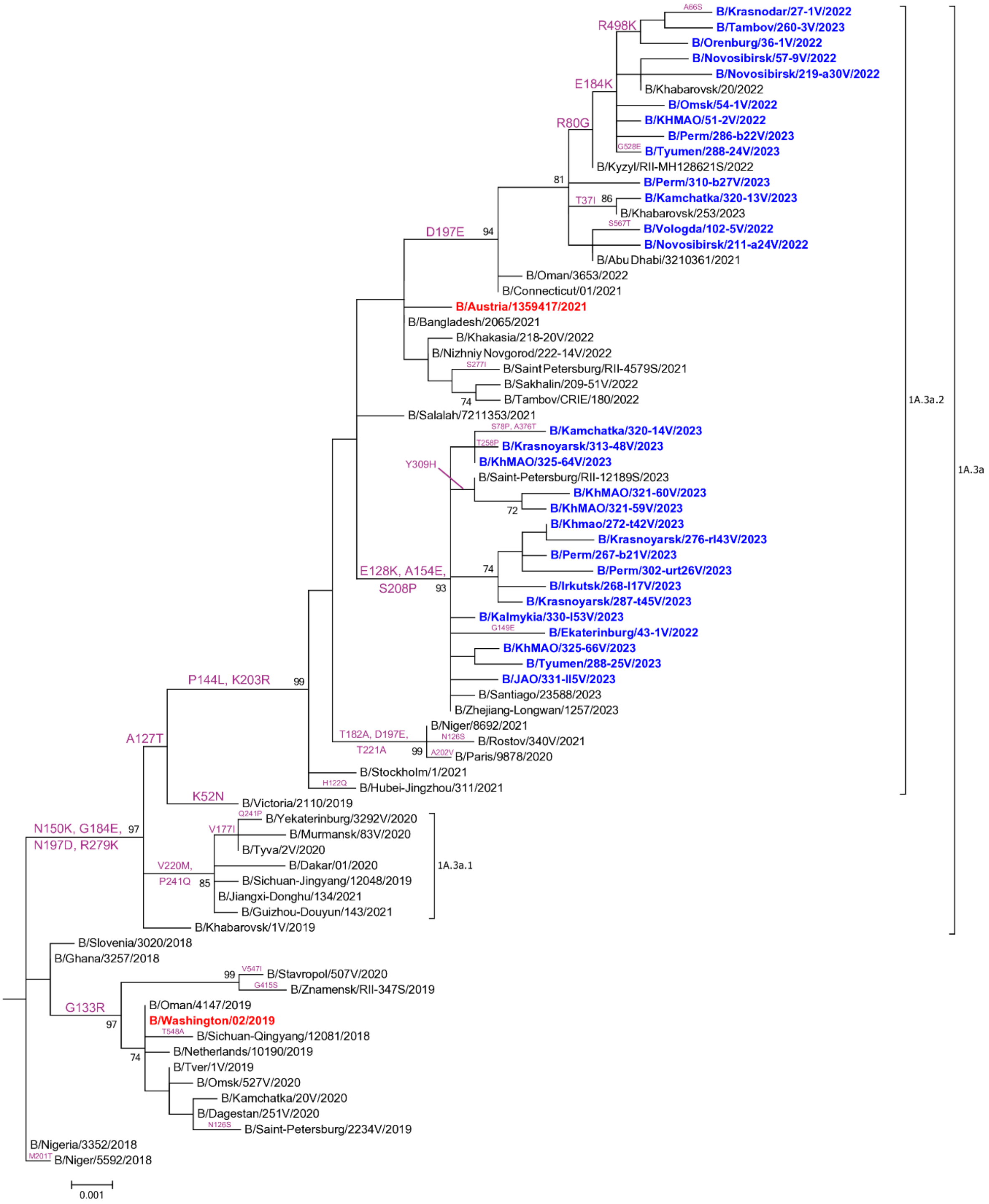

Figure S2. B/Victoria HA phylogenetic tree. A maximum likelihood phylogenetic tree of the HA gene of influenza B/Victoria viruses analyzed in this study. Viruses isolated in Russia during the 2022–2023 epidemic season are indicated in blue. Vaccine strains are indicated in red. Amino acid substitutions in HA are in purple.
