# Supplementary material for: An Investigation of Severe Influenza Cases in Russia during the 2022–2023 Epidemic Season and an Analysis of HA-D222G/N Polymorphism in Newly Emerged and Dominant Clade 6B.1A.5a.2a A(H1N1)pdm09 Viruses
Source: Pathogens. 2023 Dec 19;13(1):1. doi: 10.3390/pathogens13010001 (PMC10819184; doi:10.3390/pathogens13010001)
Supplement: Supplementary file 1 [file pathogens-13-00001-s001.zip › Supplementary Tables S1,S2,S3,S4,S5,S6,S7.pdf]

Table S1. HIA assay results for individual A(H1N1)pdm09 isolates.

(A) The table shows fold change between homologous ferret antiserum titre and titre determined in HIA with tested A(H1N1)pdm09 strains isolated in Russia in 2022-2023 flu season.

№	Tested strain	Flu vaccination status[a]	Specimen collection date	Clade	Passage history	Fold change between homologous antiserum titre and titre determined in HIA with tested strain: Ferret antisera against:			HA amino acid substitutions[b]
						A/Victoria/ 2570/2019 (egg) 5a.2	A/Wisconsin/ 588/2019 (cell culture) 5a.2	A/Guangdong-Maonan/ SWL1536/2019 (egg) 5a.1	
1	A/Altai Krai/193-l7V/2022	Unk.	20.12.2022	5a.2a	MD1[c]	0.5	0.25	16	I418V
2	A/Belgorod/73-6V/2022	NV	11.11.2022	5a.2a	MD2	0.5	0.25	16	I418V
3	A/Buryatia/106-6V/2022	NV	19.11.2022	5a.2a.1	MD2	1	0.25	64	
4	A/Cheboksary/293-t2V/2023	Unk.	13.02.2023	5a.2a	MD1	0.25	0.25	ND[d]	I418V, V19I, T391K
5	A/Chelyabinsk/307-t17V/2023	NV	08.01.2023	5a.2a	MD1	1	0.5	16	I418V, D222N
6	A/Chelyabinsk/322-l20V/2023	NV	13.01.2023	5a.2a	MD1	1	0.5	16	I418V, D222N
7	A/Ekaterinburg/111-l13V/2023	Unk.	25.11.2022	5a.2a	MD1	2	0.5	16	I418V, V47I, D187V
8	A/Ekaterinburg/225-17V/2022	V	20.12.2022	5a.2a	MD1	0.5	0.25	ND	I418V, S83P
9	A/HMAO/133-lp5V/2023	NV	30.11.2022	5a.2a	MD1	1	0.25	32	I418V
10	A/HMAO/207-20V/2023	V	10.12.2022	5a.2a	MD1	0.5	0.25	ND	I418V
11	A/HMAO/207-b24V/2022	NV	29.12.2022	5a.2a	MD1	1	0.5	16	I418V
12	A/HMAO/207-l23V/2022	NV	29.12.2022	5a.2a	MD1	1	0.25	16	I418V
13	A/Irkutsk/236-l15V/2023	NV	28.12.2022	5a.2a	MD1	0.5	0.25	16	I418V
14	A/Kaliningrad/101-rl7V/2022	NV	28.11.2022	5a.2a	MD1	0.5	0.25	ND	I418V, D222N
15	A/Kaliningrad/217-lps10V/2022	NV	21.12.2022	5a.2a	MD1	1	0.25	32	I418V, I214V, D222N

16	A/Kaliningrad/243-ls14V/2023	NV	23.12.2022	5a.2a	MD1	1	0.25	16	I418V, D222N
17	A/Kaliningrad/243-ls15V/2023	Unk.	27.12.2022	5a.2a	MD1	1	0.25	16	I418V
18	A/Kaliningrad/252-tb19V/2023	NV	10.01.2023	5a.2a	MD1	1	0.25	16	I418V
19	A/Khabarovsk/95-10V/2022	V	28.11.2022	5a.2a	MD1	0.5	0.25	ND	I418V, Q293R
20	A/KHMAO/207-t22V/2022	NV	16.12.2022	5a.2a	MD2	1	0.5	16	I418V, D222G
21	A/KhMAO/271-37V/2023	V	01.02.2023	5a.2a	MD1	0.5	0.25	ND	I418V
22	A/KhMAO/271-39V/2023	V	01.02.2023	5a.2a	MD1	0.5	0.25	ND	I418V, D269N
23	A/Krasnoyarsk/119-ll10V/2022	NV	02.12.2022	5a.2a	MD1	1	0.5	32	I418V, D222D/N
24	A/Krasnoyarsk/176-18V/2022	V	21.12.2022	5a.2a	MD1	0.25	0.25	ND	I418V
25	A/Krasnoyarsk/201-t27V/2022	Unk.	19.12.2022	5a.2a	MD1	0.25	0.25	32	I418V
26	A/Krasnoyarsk/213am28V/2022	Unk.	18.12.2022	5a.2a	MD1	1	0.25	16	I418V, V173I
27	A/Krasnoyarsk/230-ll31V/2023	NV	29.12.2022	5a.2a	MD1	0.5	0.25	ND	I418V
28	A/Krasnoyarsk/253-ll36V/2023	NV	10.01.2023	5a.2a	MD1	1	0.5	16	I418V, A261V
29	A/Murmansk/197-l6V/2022	NV	19.12.2022	5a.2a	MD1	1	0.5	ND	I418V, K302R, D222N
30	A/Novosibirsk/192-t21V/2022	Unk.	19.12.2022	5a.2a	MD2	8	2	>64	I418V, S190R
31	A/Novosibirsk/198-t22V/2022	Unk.	21.12.2022	5a.2a	MD2	1	0.5	32	I418V, D222N
32	A/Novosibirsk/218-sec27V/2022	Unk.	09.12.2022	5a.2a	MD1	1	0.25	16	I418V, D222G
33	A/Novosibirsk/69-12V/2022	Unk.	14.11.2022	5a.2a	MD2	0.5	0.25	16	I418V
34	A/Omsk/138-l7V/2022	NV	06.12.2022	5a.2a	MD1	0.5	0.25	32	I418V, V525M, D222N
35	A/Omsk/203-l8V/2022	NV	22.12.2022	5a.2a	MD1	0.5	0.25	16	I418V, Q293R, D222N
36	A/Omsk/295-t12V/2023	NV	15.02.2023	5a.2a	MD1	0.5	0.5	16	I418V
37	A/Omsk/337-l14V/2023	NV	11.04.2023	5a.2a	MD1	1	0.5	16	I418V, S326P
38	A/Orenburg/255-l14V/2023	NV	09.01.2023	5a.2a	MD1	1	0.25	16	I418V

39	A/Pskov/231-l35V/2023	Unk.	26.12.2022	5a.2a	MD1	1	0.25	16	I418V, N370D, D222N
40	A/Pskov/231-l36V/2023	Unk.	26.12.2022	5a.2a	MD1	1	0.5	16	I418V, V152I
41	A/Russia/183-9V/2022	V	15.12.2022	5a.2a	MD1	0.5	0.25	ND	I418V
42	A/Russia/319-l41V/2023	NV	18.01.2023	5a.2a	MD1	1	0.5	16	I418V, D35G, D222N
43	A/Saint-Petersburg/182-b11V/2023	Unk.	13.12.2022	5a.2a	MD1	0.5	0.25	ND	I418V, R113K, T376A, D222D/N
44	A/Sakha/164-t7V/2023	Unk.	12.12.2022	5a.2a	MD1	0.5	0.125	8	I418V
45	A/Saratov/250-rl14V/2023	Unk.	12.01.2023	5a.2a	MD1	1	0.25	16	I418V
46	A/Stavropol/233-l9V/2023	NV	18.12.2022	5a.2a	MD1	1	0.25	16	I418V
47	A/Tatarstan/174-ll13V/2022	NV	08.12.2022	5a.2a	MD1	0.5	0.125	16	I418V
48	A/Tomsk/240-l7V/2023	Unk.	12.01.2023	5a.2a	MD1	0.5	0.25	ND	I418V
49	A/Tver/107-t2V/2022	NV	28.11.2022	5a.2a	MD1	1	0.25	16	I418V
50	A/Tyumen/185-l13V/2022	NV	15.12.2022	5a.2a	MD1	1	0.25	16	I418V, D222N
51	A/Tyumen/234-l15V/2023	NV	11.01.2023	5a.2a	MD1	2	0.5	8	I418V, D222N
52	A/Vladimir/163-l2V/2022	NV	05.12.2022	5a.2a	MD1	0.5	0.25	ND	I418V, D222N
53	A/Vologda/168-l8V/2023	NV	06.12.2022	5a.2a	MD1	1	0.25	16	I418V, E283K
54	A/Vologda/189-t10V/2022	NV	13.12.2022	5a.2a	MD1	0.5	0.25	16	I418V
55	A/Yarosavl/301-l12V/2023	NV	18.02.2023	5a.2a	MD1	0.5	0.25	16	I418V
56	A/Yarosavl/301-t14V/2023	NV	18.02.2023	5a.2a	MD1	0.5	0.5	16	I418V
57	A/Yaroslavl/128-pl1V/2022	NV	06.12.2022	5a.2a	MD1	0.5	0.25	16	I418V, N455D, Y468H, D222D/N
58	A/Yaroslavl/238-l11V/2023	NV	24.12.2022	5a.2a	MD1	1	0.25	16	I418V
59	A/Yaroslavl/301-l16V/2023	Unk.	18.02.2023	5a.2a	MD1	0.5	0.5	16	I418V
60	A/Yekaterinburg/111-l11V/2022	Unk.	21.11.2022	5a.2a	MD1	1	0.25	16	I418V

61	A/Kemerovo/154-10V/2022	V	07.12.2022	ND	MD1	0.5	0.25	ND	ND
62	A/Kemerovo/154-7V/2022	V	05.12.2022	ND	MD1	0.5	0.25	ND	ND
63	A/Kemerovo/154-8V/2022	V	07.12.2022	ND	MD1	0.5	0.25	16	ND
64	A/Kemerovo/154-9V/2022	V	07.12.2022	ND	MD1	0.5	0.25	ND	ND
65	A/KhMAO/179-15V/2022	V	13.12.2022	ND	MD1	0.5	0.25	ND	ND
66	A/Kurgan/145-9V/2022	V	30.11.2022	ND	MD1	0.5	0.5	ND	ND
67	A/Saratov/181-11V/2022	V	13.12.2022	ND	MD1	0.5	0.25	ND	ND
68	A/Saratov/181-12V/2022	V	13.12.2022	ND	MD1	0.5	0.25	ND	ND
69	A/Stavropol/124-6V/2022	V	28.11.2022	ND	MD1	0.25	0.25	ND	ND

[a] – Flu vaccination status: V – vaccinated, NV – not vaccinated, Unk. - unknown;

[b] – non clade-specific HA amino acid substitutions compared to A/Victoria/2570/2019. In addition to the substitutions indicated in the table, all viruses from the 5a.2a and 5a.2a.1 clades have a subset of the clade-specific substitutions (K54Q, A186T, Q189E, E224A, R259K, K308R); virus from the 5a.2a.1 clade also have additional 5a.2a.1-clade specific substitutions P137S, K142R, D260E, T277A, E356D, I418V and N451H, compared to A/Victoria/2570/2019.

[c] – MD – MDCK cell culture;

[d] – ND – not determined.

(B) Characterization of influenza A(H1N1)pdm09 viruses in HIA with turkey red blood cells (1).

Virus	Clade	Passage history	Collection date	Reverse HIA titers with ferret antisera raised against:		
				A/Guangdong-Maonan/SWL1536/2019	A/Victoria/2570/2019	A/Wisconsin/588/2019
				egg	egg	cell
				fer. 11/20, FCI	fer. 27/20, FCI	fer. 25/20, FCI
				6B.1A.5a.1	6B.1A.5a.2	6B.1A.5a.2
**Reference strains**						
A/Guangdong-Maonan/SWL1536/2019	6B.1A.5a.1	Ex/E1	17.06.2019	**5120**	<160	<320
A/Victoria/2570/2019	6B.1A.5a.2	E4/E2/E1	22.11.2019	640	**1280**	5120
A/Wisconsin/588/2019	6B.1A.5a.2	C2/SIAT1/MD2/MD1	19.12.2019	1280	320	**2560**
**Tested strains**						
A/Novosibirsk/198-t22V/2022	6B.1A.5a.2a	MDCK2	21.12.2022	160	1280	5120
A/Krasnoyarsk/119-Il10V/2022	6B.1A.5a.2a	MDCK1	02.12.2022	160	1280	5120
A/Buryatia/106-6V/2022	6B.1A.5a.2a.1	MDCK2	19.11.2022	80	1280	10240
A/Kemerovo/154-8V/2022		MDCK1	07.12.2022	320	2560	10240
A/KHMAO/207-t22V/2022	6B.1A.5a.2a	MDCK2	16.12.2022	320	1280	5120
A/Belgorod/73-6V/2022	6B.1A.5a.2a	MDCK2	11.11.2022	320	2560	10240
A/Novosibirsk/69-12V/2022	6B.1A.5a.2a	MDCK2	14.11.2022	320	2560	10240
A/Ekaterinburg/111-l13V/2023	6B.1A.5a.2a	MDCK1	25.11.2022	320	640	5120
A/Novosibirsk/192-t21V/2022	6B.1A.5a.2a	MDCK2	19.12.2022	<80	160	1280

Fold change relative to the homologous titer:

(green)	≤ 4
(orange)	≥ 8

NA – not assessed

Characterization of influenza A(H1N1)pdm09 viruses in HIA with turkey red blood cells (2).

Virus	Clade	Passage history	Collection date	Reverse HIA titers with ferret antisera raised against:	
				A/Victoria/2570/2019	A/Wisconsin/588/2019
				egg	cell
				fer. 27/20, FCI	fer. 25/20, FCI
				6B.1A.5a.2	6B.1A.5a.2
**Reference strains**					
A/Victoria/2570/2019	6B.1A.5a.2	E4/E2/E1	22.11.2019	**640**	2560
A/Wisconsin/588/2019	6B.1A.5a.2	C2/SIAT1/MD2/MD1	19.12.2019	320	**1280**
**Tested strains**					
A/Saint-Petersburg/182-b11V/2023	6B.1A.5a.2a	MDCK1	13.12.2022	1280	5120
A/Vladimir/163-l2V/2022	6B.1A.5a.2a	MDCK1	05.12.2022	1280	5120
A/Cheboksary/293-t2V/2023	6B.1A.5a.2a	MDCK1	13.02.2023	2560	5120
A/Murmansk/197-l6V/2022	6B.1A.5a.2a	MDCK1	19.12.2022	640	2560
A/Krasnoyarsk/230-ll31V/2023	6B.1A.5a.2a	MDCK1	29.12.2022	1280	5120
A/Tomsk/240-l7V/2023	6B.1A.5a.2a	MDCK1	12.01.2023	1280	5120
A/Buryatia/106-6V/2022	6B.1A.5a.2a.1	MDCK1	19.11.2022	1280	5120
A/KhMAO/271-39V/2023	6B.1A.5a.2a	MDCK1	01.02.2023	1280	5120
A/Saratov/181-11V/2022	Not determined	MDCK1	13.12.2022	1280	5120
A/KhMAO/271-37V/2023	6B.1A.5a.2a	MDCK1	01.02.2023	1280	5120
A/Khabarovsk/95-10V/2022	6B.1A.5a.2a	MDCK1	28.11.2022	1280	5120
A/Stavropol/124-6V/2022	Not determined	MDCK1	28.11.2022	2560	5120
A/Kemerovo/154-9V/2022	Not determined	MDCK1	07.12.2022	1280	5120
A/Kurgan/145-9V/2022	Not determined	MDCK1	30.11.2022	1280	2560
A/Kemerovo/154-10V/2022	Not determined	MDCK1	07.12.2022	1280	5120
A/KhMAO/179-15V/2022	Not determined	MDCK1	13.12.2022	1280	5120
A/HMAO/207-20V/2023	6B.1A.5a.2a	MDCK1	10.12.2022	1280	5120
A/Krasnoyarsk/176-18V/2022	6B.1A.5a.2a	MDCK1	21.12.2022	2560	5120
A/Ekaterinburg/225-17V/2022	6B.1A.5a.2a	MDCK1	20.12.2022	1280	5120
A/Russia/183-9V/2022	Not determined	MDCK1	15.12.2022	1280	5120
A/Saratov/181-12V/2022	Not determined	MDCK1	13.12.2022	1280	5120
A/Kemerovo/154-7V/2022	Not determined	MDCK1	05.12.2022	1280	5120
A/Kaliningrad/101-ll7V/2022	6B.1A.5a.2a	MDCK1	28.11.2022	1280	5120
A/Novosibirsk/198-t22V/2022	6B.1A.5a.2a	MDCK1	21.12.2022	1280	5120
A/KHMAO/207-t22V/2022	6B.1A.5a.2a	MDCK1	16.12.2022	1280	NA
A/Belgorod/73-6V/2022	6B.1A.5a.2a	MDCK1	11.11.2022	2560	NA
A/Ekaterinburg/111-l13V/2023	6B.1A.5a.2a	MDCK1	25.11.2022	640	NA
A/Sakha/164-t7V/2023	6B.1A.5a.2a	MDCK1	12.12.2022	1280	NA
A/Tatarstan/174-ll13V/2022	6B.1A.5a.2a	MDCK1	08.12.2022	1280	NA
A/Altai Krai/193-l7V/2022	6B.1A.5a.2a	MDCK1	20.12.2022	1280	NA
A/Yaroslavl/128-pl1V/2022	6B.1A.5a.2a	MDCK1	06.12.2022	1280	NA
A/Omsk/203-l8V/2022	6B.1A.5a.2a	MDCK1	22.12.2022	1280	NA
A/Omsk/138-l7V/2022	6B.1A.5a.2a	MDCK1	06.12.2022	1280	NA
A/Krasnoyarsk/201-t27V/2022	6B.1A.5a.2a	MDCK1	19.12.2022	2560	NA

Fold change relative to the homologous titer:

(green)	≤ 4
(orange)	≥ 8

NA – not assessed

Characterization of influenza A(H1N1)pdm09 viruses in HIA with turkey red blood cells (3)

Virus	Clade	Passage history	Collection date	Reverse HIA titers with ferret antisera raised against:		
				A/Guangdong-Maonan/SWL1536/2019	A/Victoria/2570/2019	A/Wisconsin/588/2019
				egg	egg	cell
				fer. 11/20, FCI	fer. 27/20, FCI	fer. 25/20, FCI
				6B.1A.5a.1	6B.1A.5a.2	6B.1A.5a.2
**Reference strains**						
A/Guangdong-Maonan/SWL1536/2019	6B.1A.5a.1	Ex/E1	17.06.2019	**5120**	NA	NA
A/Victoria/2570/2019	6B.1A.5a.2	E4/E2/E1	22.11.2019	640	**1280**	2560
A/Wisconsin/588/2019	6B.1A.5a.2	C2/SIAT1/MD2/MD1	19.12.2019	1280	320	**1280**
**Tested strains**						
A/Pskov/231-l36V/2023	6B.1A.5a.2a	MDCK1	26.12.2022	320	1280	2560
A/Krasnoyarsk/213am28V/2022	6B.1A.5a.2a	MDCK1	18.12.2022	320	1280	5120
A/Yaroslavl/238-l11V/2023	6B.1A.5a.2a	MDCK1	24.12.2022	320	1280	5120
A/Kaliningrad/243-ls14V/2023	6B.1A.5a.2a	MDCK1	23.12.2022	320	1280	5120
A/Tver/107-t2V/2022	6B.1A.5a.2a	MDCK1	28.11.2022	320	1280	5120
A/Yekaterinburg/111-l11V/2022	6B.1A.5a.2a	MDCK1	21.11.2022	320	1280	5120
A/Vologda/189-t10V/2022	6B.1A.5a.2a	MDCK1	13.12.2022	320	2560	5120
A/Pskov/231-l35V/2023	6B.1A.5a.2a	MDCK1	26.12.2022	320	1280	5120
A/Kaliningrad/217-lps10V/2022	6B.1A.5a.2a	MDCK1	21.12.2022	160	1280	5120
A/HMAO/207-l23V/2022	6B.1A.5a.2a	MDCK1	29.12.2022	320	1280	5120
A/Kaliningrad/252-tb19V/2023	6B.1A.5a.2a	MDCK1	10.01.2023	320	1280	5120
A/Tyumen/185-l13V/2022	6B.1A.5a.2a	MDCK1	15.12.2022	320	1280	5120
A/HMAO/133-lp5V/2023	6B.1A.5a.2a	MDCK1	30.11.2022	160	1280	5120
A/Tyumen/234-l15V/2023	6B.1A.5a.2a	MDCK1	11.01.2023	640	640	2560
A/Saratov/250-rl14V/2023	6B.1A.5a.2a	MDCK1	12.01.2023	320	1280	5120
A/Stavropol/233-l9V/2023	6B.1A.5a.2a	MDCK1	18.12.2022	320	1280	5120
A/Kaliningrad/243-ls15V/2023	6B.1A.5a.2a	MDCK1	27.12.2022	320	1280	5120
A/Orenburg/255-l14V/2023	6B.1A.5a.2a	MDCK1	09.01.2023	320	1280	5120
A/Vologda/168-l8V/2023	6B.1A.5a.2a	MDCK1	06.12.2022	320	1280	5120
A/Irkutsk/236-l15V/2023	6B.1A.5a.2a	MDCK1	28.12.2022	320	2560	5120
A/Novosibirsk/218-sec27V/2022	6B.1A.5a.2a	MDCK1	09.12.2022	320	1280	5120

Fold change relative to the homologous titer:

(green)	≤ 4
(orange)	≥ 8

Characterization of influenza A(H1N1)pdm09 viruses in HIA with turkey red blood cells (4)

Virus	Clade	Passage history	Collection date	Reverse HIA titers with ferret antisera raised against:		
				A/Guangdong-Maonan/SWL1536/2019	A/Victoria/2570/2019	A/Wisconsin/588/2019
				egg	egg	cell
				fer. 11/20, FCI	fer. 27/20, FCI	fer. 25/20, FCI
				6B.1A.5a.1	6B.1A.5a.2	6B.1A.5a.2
**Reference strains**						
A/Guangdong-Maonan/SWL1536/2019	6B.1A.5a.1	Ex/E1	17.06.2019	**5120**	<320	<640
A/Victoria/2570/2019	6B.1A.5a.2	E4/E2/E1	22.11.2019	640	**1280**	5120
A/Wisconsin/588/2019	6B.1A.5a.2	C2/SIAT1/MD2/MD1	19.12.2019	1280	640	**2560**
**Tested strains**						
A/Chelyabinsk/307-t17V/2023	6B.1A.5a.2a	MDCK1	08.01.2023	320	1280	5120
A/Yaroslavl/301-l16V/2023	6B.1A.5a.2a	MDCK1	18.02.2023	320	2560	5120
A/Yarosavl/301-l12V/2023	6B.1A.5a.2a	MDCK1	18.02.2023	320	2560	10240
A/Yarosavl/301-t14V/2023	6B.1A.5a.2a	MDCK1	18.02.2023	320	2560	5120
A/Krasnoyarsk/253-ll36V/2023	6B.1A.5a.2a	MDCK1	10.01.2023	320	1280	5120
A/Omsk/295-t12V/2023	6B.1A.5a.2a	MDCK1	15.02.2023	320	2560	5120
A/HMAO/207-b24V/2022	6B.1A.5a.2a	MDCK1	29.12.2022	320	1280	5120
A/Omsk/337-l14V/2023	6B.1A.5a.2a	MDCK1	11.04.2023	320	1280	5120
A/Chelyabinsk/322-l20V/2023	6B.1A.5a.2a	MDCK1	13.01.2023	320	1280	5120
A/Russia/319-l41V/2023		MDCK1	18.01.2023	320	1280	5120

Fold change relative to the homologous titer:

(green)	≤ 4
(orange)	≥ 8

Characterization of influenza A(H1N1)pdm09 viruses in HIA with turkey red blood cells (5)

Virus	Clade	Passage history	Collection date	Reverse HIA titers with ferret antisera raised against:	
				A/Guangdong-Maonan/SWL1536/2019	A/Wisconsin/588/2019
				egg	cell
				fer. 11/20, FCI	fer. 25/20, FCI
				6B.1A.5a.1	6B.1A.5a.2
**Reference strains**					
A/Guangdong-Maonan/SWL1536/2019	6B.1A.5a.1	Ex/E1	17.06.2019	**5120**	NA
A/Wisconsin/588/2019	6B.1A.5a.2	C2/SIAT1/MD2/MD1	19.12.2019	NA	**1280**
**Tested strains**					
A/Sakha/164-t7V/2023	6B.1A.5a.2a	MDCK1	12.12.2022	640	10240
A/Tatarstan/174-Il13V/2022	6B.1A.5a.2a	MDCK1	08.12.2022	320	10240
A/Novosibirsk/198-t22V/2022	6B.1A.5a.2a	MDCK1	21.12.2022	320	5120
A/Altai Krai/193-l7V/2022	6B.1A.5a.2a	MDCK1	20.12.2022	320	5120
A/Buryatia/106-6V/2022	6B.1A.5a.2a.1	MDCK1	19.11.2022	80	5120
A/KHMAO/207-t22V/2022	6B.1A.5a.2a	MDCK1	16.12.2022	640	5120
A/Yaroslavl/128-pl1V/2022	6B.1A.5a.2a	MDCK1	06.12.2022	320	5120
A/Ekaterinburg/111-l13V/2023	6B.1A.5a.2a	MDCK1	25.11.2022	640	2560
A/Omsk/203-l8V/2022	6B.1A.5a.2a	MDCK1	22.12.2022	320	5120

Fold change relative to the homologous titer:

(green)	≤ 4
(orange)	≥ 8

Characterization of influenza A(H1N1)pdm09 viruses in HIA with turkey red blood cells (6)

Virus	Clade	Passage history	Collection date	Reverse HIA titers with ferret antisera raised against:		
				A/Guangdong-Maonan/SWL1536/2019	A/Wisconsin/588/2019	A/Darwin/9/2021
				egg	cell	egg
				fer. 11/20, FCI	fer. 25/20, FCI	fer. 38/21, FCI
				6B.1A.5a.1	6B.1A.5a.2	A(H3N2)
**Reference strains**						
A/Guangdong-Maonan/SWL1536/2019	6B.1A.5a.1	Ex/E1	17.06.2019	**10240**	160	<20
A/Hawaii/70/2019	6B.1A.5a.1	Cx/MD1	05.10.2019	10240	160	<20
A/Wisconsin/588/2019	6B.1A.5a.2	C2/SIAT1/MD2/MD1	19.12.2019	1280	**2560**	<20
A/Darwin/9/2021		E3/E2/E1	17.04.2021	NA	NA	**2560**
**Tested strains**						
A/Novosibirsk/69-12V/2022	6B.1A.5a.2a	MDCK1	14.11.2022	320	10240	<20
A/Omsk/138-b7V/2022	6B.1A.5a.2a	MDCK1	06.12.2022	320	10240	<20
A/Belgorod/73-6V/2022	6B.1A.5a.2a	MDCK1	11.11.2022	320	10240	<20
A/Krasnoyarsk/201-t27V/2022	6B.1A.5a.2a	MDCK1	19.12.2022	320	10240	<20

Fold change relative to the homologous titer:

(green)	≤ 4
(orange)	≥ 8

NA – not assessed

Table S2. Summary table of the A(H1N1)pdm09 HIA results.

The table shows fold change between homologous ferret antiserum titre and titre determined in HIA with tested A(H1N1)pdm09 strains isolated in Russia in 2022-2023 flu season (summary table).

**Fold change between homologous antiserum titre and titre determined in HIA with tested A(H1N1)pdm09 isolate**	**Number and proportion of A(H1N1)pdm09 isolates with the specified fold change of HIA titre. Ferret antisera raised against:**		
	**A/Victoria/ 2570/ 2019 (egg[a]) 5a.2[b]**	**A/Wisconsin/588/2019 (c.c.[c]) 5a.2**	**A/Guangdong-Maonan/SWL1536/2019 (egg) 5a.1**
0.125	0 (0%)	2 (2.9%)	0 (0%)
0.25	4 (5.8%)	49 (71%)	0 (0%)
0.5	33 (47.8%)	17 (24.6%)	0 (0%)
1	29 (42%)	0 (0%)	0 (0%)
2	2 (2.9%)	1 (1.4%)	0 (0%)
8	1 (1.4%)	0 (0%)	2 (4.3%)
16	0 (0%)	0 (0%)	37 (78.7%)
32	0 (0%)	0 (0%)	6 (12.8%)
64	0 (0%)	0 (0%)	1 (2.1%)
>64	0 (0%)	0 (0%)	1 (2.1%)
**Number and proportion of isolates within the specified range of fold change:**			
0.125 - 2	68 (98.6%)	69 (100%)	0 (0%)
≥8	1 (1.4%)	0 (0%)	47 (100%)
**Total number of tested isolates:**	69 (100%)	69 (100%)	47 (100%)

[a] – egg-grown virus was used to infect ferret to raise antiserum;
[b] – clade of the virus which was used to infect ferret to raise antiserum;
[c] – cell culture (c.c.) grown virus was used to infect ferret to raise antiserum.

Table S3. HIA assay results for the A(H3N2) isolate.

Analysis of A(H3N2) virus isolated in Russia in 2022-2023 in HIA (hemagglutination inhibition assay) with reference ferret antisera. Turkey red blood cells, 20nM oseltamivir

**Virus**	**Clade**	**Passage history**	**Specimen collection date**	**HIA antiserum titres. Ferret antisera raised against:**		
				**A/Cambodia/ e0826360/2020**	**A/Darwin/ 9/2021**	**A/Stockholm/ 5/2021**
				egg[a]	egg	cell[b]
				fer. 13/21, FCI[c]	fer. 38/21, FCI	fer. 35/21, FCI
				1a[d]	2a	2a
**Reference strains**						
A/Cambodia/e0826360/2020	1a	E5 /E2/E1	16.07.2020	**5120**	640	640
A/Darwin/9/2021	2a	E3/E2 / E1	17.04.2021	640	**2560**	1280
A/Stockholm/5/2021	2a	SIAT0/SIAT3/MDCK1	16.04.2021	160	2560	**1280**
**Tested strain**						
A/Khabarovsk/95-5V/2022	2b	MDCK2	21.11.2022	320	640	640

[a] – egg-grown virus was used to infect ferret to raise antiserum;
[b] – cell-grown virus was used to infect ferret to raise antiserum;
[c] – catalogue number of reference ferret antiserum;
[d] – clade of the virus which was used to infect ferret.

Table S4. HIA assay results for individual B/Victoria isolates.

(A) The table shows fold change between homologous ferret antiserum titre and titre determined in HIA with tested A(H1N1)pdm09 strains isolated in Russia in 2022-2023 flu season.

№	Tested strains	Flu vaccination status[a]	Sample collection date	Clade	Passage history	Fold change between homologous antiserum titre and the titre determined in HIA with tested strain: Ferret antiserum against:				HA amino acid substitutions compared to B/Austria/ 1359417/2021
						B/Washington/ 02/ 2019 (c.c.[b]) V1A.3	B/Washington/ 02/ 2019 (egg) V1A.3	B/Austria/ 1359417/ 2021 (c.c.) V1A.3a.2	B/Austria/ 1359417/ 2021, isolate 1 (egg) V1A.3a.2	
1	B/Perm/286-b22V/2023	NV	03.02.2023	V1A.3a.2	MD1[c]	2	16	1	1	D197E, R80G, E184K
2	B/Perm/302-urt26V/2023	NV	13.02.2023	V1A.3a.2	MD1	>4[d]	16	1	1	E128K, A154E, S208P
3	B/JAO/331-ll5V/2023	NV	04.04.2023	V1A.3a.2	MD1	>4	16	1	1	E128K, A154E, S208P
4	B/Perm/310-b27V/2023	NV	01.03.2023	V1A.3a.2	MD1	4	16	1	1	D197E, E128K
5	B/Orenburg/36-1V/2022	NV	21.10.2022	V1A.3a.2	MD1	2	16	1	1	D197E, R80G, E184K, R498K
6	B/KhMAO/329-76V/2023	V	24.03.2023	ND[e]	MD1	4	16	1	1	Н.И.
7	B/KhMAO/328-71V/2023	V	29.03.2023	ND	MD1	>4	16	1	1	Н.И.
8	B/Krasnodar/27-1V/2022	Unk.	24.10.2022	V1A.3a.2	MD1	1	8	1	0.5	D197E, R80G, E184K, A66S, R498K
9	B/Tyumen/288-24V/2023	NV	02.02.2023	V1A.3a.2	MD1	1	8	1	0.5	D197E, R80G, E184K
10	B/KhMAO/303-51V/2023	NV	14.02.2023	V1A.3a.2	MD1	>2	8	1	1	E128K, A154E, S208P, V87I, T121N
11	B/Yekaterinburg/281-19V/2023	NV	25.01.2023	ND	MD1	1	8	1	0.5	Н.И.
12	B/Kalmykia/330-l53V/2023	NV	27.03.2023	V1A.3a.2	MD1	>2	8	1	0.5	E128K, A154E, S208P

13	B/KhMAO/325-64V/2023	V	20.03.2023	V1A.3a.2	MD1	>2	8	1	0.5	E128K, A154E, S208P, Y309H
14	B/Yekaterinburg/281-20V/2023	NV	25.01.2023	ND	MD1	>2	8	1	0.5	Н.И.
15	B/KhMAO/324-63V/2023	NV	18.03.2023	V1A.3a.2	MD1	>2	8	1	0.5	E128K, A154E, S208P, Y309H
16	B/KhMAO/339-78V/2023	V	10.04.2023	ND	MD1	>2	8	1	1	Н.И.
17	B/Lipetsk/316-1.1V/2023	NV	09.03.2023	ND	MD1	>2	8	1	0.5	Н.И.
18	B/Omsk/54-1V/2022	NV	15.11.2022	V1A.3a.2	MD1	1	8	1	0.5	D197E, R80G, E184K
19	B/Novosibirsk/57-9V/2022	NV	08.11.2022	V1A.3a.2	MD1	>2	16	1	0.5	D197E, R80G, E184K, E128K
20	B/Vologda/102-5V/2022	NV	18.11.2022	V1A.3a.2	MD1	>2	8	1	0.5	D197E, S567T
21	B/KhMAO/325-66V/2023	V	18.03.2023	V1A.3a.2	MD1	>2	8	1	1	E128K, A154E, S208P
22	B/KhMAO/325-65V/2023	V	20.03.2023	V1A.3a.2	MD1	>2	8	1	1	E128K, A154E, S208P, Y309H
23	B/Tyumen/288-25V/2023	NV	09.02.2023	V1A.3a.2	MD1	>2	8	1	1	E128K, A154E, S208P, G528E

[a] – Flu vaccination status: V – vaccinated, NV – not vaccinated, Unk. - unknown;

[b] – c.c. – antiserum against the cell culture-grown virus;

[c] – MD – MDCK cell culture;

[d] – titre fold difference was not determined precisely because of the low homologous titre of anti-B/Washington/02/2019 (c.c.) (1/40);

[e] – ND – not determined.

(B) Characterization of influenza B/Victoria viruses in HIA with turkey red blood cells (1).

Virus	Clade	Passage history	Collection date	Reverse HIA titers with ferret antisera raised against:				
				B/Washington/ 02/2019	B/Washington/ 02/2019	B/Austria/ 1359417/2021	B/Austria/ 1359417/2021, isolate 1	B/Phuket/ 3073/2013
				cell	egg	cell	egg	egg
				fer. 37/19, FCI*	fer. 38/19, FCI	fer. 22/21, FCI	fer. 15/21, FCI	fer. 22/18, FCI
				B/Victoria	B/Victoria	B/Victoria	B/Victoria	B/Yamagata
				V1A.3	V1A.3	V1A.3a.2	V1A.3a.2	
**Reference strains**								
B/Washington/02/2019	V1A.3	Cx/MD1	19.01.2019	**80**	640	20	80	<40
B/Washington/02/2019	V1A.3	Ex/E1	19.01.2019	80	**1280**	20	80	<40
B/Austria/1359417/2021	V1A.3a.2	SIAT1/MD4/MD1	09.01.2021	40	80	**1280**	2560	<40
B/Austria/1359417/2021	V1A.3a.2	Ex/E1	09.01.2021	40	80	640	**2560**	<40
B/Phuket/3073/2013		Ex/E1		<20	<20	<20	40	**640**
**Tested strains**								
B/Perm/286-b22V/2023	V1A.3a.2	MDCK1	03.02.2023	40	80	1280	2560	<40
B/Perm/302-urt26V/2023	V1A.3a.2	MDCK1	13.02.2023	<20	80	1280	2560	<40
B/JAO/331-ll5V/2023	V1A.3a.2	MDCK1	04.04.2023	<20	80	1280	2560	<40
B/Perm/310-b27V/2023	V1A.3a.2	MDCK1	01.03.2023	20	80	1280	2560	<40
B/Orenburg/36-1V/2022	V1A.3a.2	MDCK1	21.10.2022	40	80	1280	2560	<40
B/KhMAO/329-76V/2023	Not determined	MDCK1	24.03.2023	20	80	1280	2560	<40
B/KhMAO/328-71V/2023	Not determined	MDCK1	29.03.2023	<20	80	1280	2560	<40

* - Catalogue number of ferret antisera provided by the Francis Crick Institute (FCI) in 2021

Fold change relative to the homologous titer:

(green)	≤ 4
(orange)	≥ 8

Characterization of influenza B/Victoria viruses in HIA with turkey red blood cells (2).

Strain	Clade	Passage history	Collection date	Reverse HIA titers with ferret antisera raised against:			
				B/Washington/ 02/2019	B/Washington/ 02/2019	B/Austria/ 1359417/2021	B/Austria/1359 417/ 2021, isolate 1
				cell	egg	cell	egg
				fer. 37/19, FCI*	fer. 38/19, FCI	fer. 22/21, FCI	fer. 15/21, FCI
				B/Vic	B/Vic	B/Vic	B/Vic
				V1A.3	V1A.3	V1A.3a. 2	V1A.3a.2
**Reference strains**							
B/Washington/02/2019	V1A.3	Cx/MD1	19.01.2019	**40**	320	<160	<160
B/Washington/02/2019	V1A.3	Ex/E1	19.01.2019	40	**640**	<160	<160
B/Austria/1359417/2021	V1A.3a.2	SIAT1/MD4/ MD1	09.01.2021	40	80	**1280**	2560
B/Austria/1359417/2021	V1A.3a.2	Ex/E1	09.01.2021	20	40	640	**1280**
**Tested strains**							
B/Krasnodar/27-1V/2022	V1A.3a.2	MDCK1	24.10.2022	40	80	1280	2560
B/Tyumen/288-24V/2023	V1A.3a.2	MDCK1	02.02.2023	40	80	1280	2560
B/KhMAO/303-51V/2023	V1A.3a.2	MDCK1	14.02.2023	<20	80	1280	1280
B/Yekaterinburg/281-19V/2023	Not determined	MDCK1	25.01.2023	40	80	1280	2560
B/Kalmykia/330-153V/2023	V1A.3a.2	MDCK1	27.03.2023	<20	80	1280	2560
B/KhMAO/325-64V/2023	V1A.3a.2	MDCK1	20.03.2023	<20	80	1280	2560
B/Yekaterinburg/281-20V/2023	Not determined	MDCK1	25.01.2023	<20	80	1280	2560
B/KhMAO/324-63V/2023	V1A.3a.2	MDCK1	18.03.2023	<20	80	1280	2560
B/KhMAO/339-78V/2023	Not determined	MDCK1	10.04.2023	<20	80	1280	1280
B/Lipetsk/316-1.1V/2023	Not determined	MDCK1	09.03.2023	<20	80	1280	2560
B/Omsk/54-1V/2022	V1A.3a.2	MDCK1	15.11.2022	40	80	1280	2560
B/Novosibirsk/57-9V/2022	V1A.3a.2	MDCK1	08.11.2022	<20	40	1280	2560
B/Vologda/102-5V/2022	V1A.3a.2	MDCK1	18.11.2022	<20	80	1280	2560
B/KhMAO/325-66V/2023	V1A.3a.2	MDCK1	18.03.2023	<20	80	1280	1280
B/KhMAO/325-65V/2023	V1A.3a.2	MDCK1	20.03.2023	<20	80	1280	1280
B/Tyumen/288-25V/2023	V1A.3a.2	MDCK1	09.02.2023	<20	80	1280	1280

* - Catalogue number of ferret antisera provided by the Francis Crick Institute (FCI) in 2021

Fold change relative to the homologous titer:

(green)	≤ 4
(orange)	≥ 8

Table S5. Summary table of the B/Victoria HIA results.

Fold change between homologous ferret antiserum titre and titre determined in HIA with tested B/Victoria viruses isolated in Russia in 2022-2023 flu season (summary table).

**Fold change between homologous antiserum titre and titre determined in HIA with tested B/Victoria isolate**	**Number and proportion of B/Victoria isolates with the specified fold change of HIA titre. Ferret antisera raised against:**			
	Anti-B/Washington/02/2019 (c.c.[b]) V1A.3[c]	Anti-B/Washington/02/2019 (egg[d]) V1A.3	Anti-B/Austria/1359417/2021 (c.c.) V1A.3a.2	Anti-B/Austria/1359417/2021, isolate 1 (egg) V1A.3a.2
0.5	0 (0%)	0 (0%)	0 (0%)	11 (47.8%)
1	4 (17.4%)	0 (0%)	23 (100%)	12 (52.2%)
2	2 (8.7%)	0 (0%)	0 (0%)	0 (0%)
4	2 (8.7%)	0 (0%)	0 (0%)	0 (0%)
8	0 (0%)	15 (65.2%)	0 (0%)	0 (0%)
16	0 (0%)	8 (34.8%)	0 (0%)	0 (0%)
>2[a]	12 (52.2%)	0 (0%)	0 (0%)	0 (0%)
>4[a]	3 (13%)	0 (0%)	0 (0%)	0 (0%)
**Number and proportion of isolates within the specified range of fold change:**				
≤ 4	8 (34.8%)	0 (0%)	23 (100%)	23 (100%)
>4	3 (13%)	23 (100%)	0 (0%)	0 (0%)
**Total number of tested isolates:**	23 (100%)	23 (100%)	23 (100%)	23 (100%)

[a] – titre fold difference was not determined precisely because of the low homologous titre of anti-B/Washington/02/2019 (c.c.) (1/40);
[b] – c.c. – antiserum against the cell culture-grown virus;
[c] – clade of the virus which was used to infect ferret to raise antiserum;
[d] – antiserum raised against the egg-grown virus.

Table S6. Analysis of the 222D/G/N polymorphism in the A(H1N1)pdm09 virus samples (D222G/N substitutions in the major virus variant of the samples).

Virus specimen	Virus source	Specimen type	222D (GAT)	222N (AAT)	222G (GGT)	NGS coverage#
Viruses from cases with fatal outcomes						
A/Kaliningrad/101-ll7V/2022	lung	Original	16.6	**67.6**	6.3	13675
A/Tver/107-l1V/2022	lung	Original	36.2	**43.3**	15.9	13382
A/Omsk/138-b7V/2022	bronchi	Original	38	**56.7**	0.3	1486
A/Vladimir/163-l2V/2022	lung	Original	5.6	**78.1**	9.5	1508
A/Saratov/181-l13V/2022	lung	Original	0.5	**94.7**	-	56878
A/Novosibirsk/198-t22V/2022	trachea	Isolate	0.3	**95.1**	-	22388
A/Tyumen/185-l13V/2022	lung	Isolate	0.3	**94.3**	1	50856
A/Murmansk/197-l6V/2022	lung	Isolate	0.2	**95**	-	42544
A/Kaliningrad/217-lps10V/2022	lung	Isolate	0.2	**90.1**	4.9	51878
A/Omsk/203-l8V/2022	lung	Isolate	19	**72.9**	3.1	70736
A/Yaroslavl/128-pl1V/2022	lung	Isolate	0.2	**95.2**	-	28740
A/Sakhalin/210-ll58V/2022	lung	Isolate	0.3	**95.5**	-	94786
A/Krasnoyarsk/119-ll10V/2022	lung	Isolate	22.6	**71**	1.3	36424
A/Tyumen/234-l15V/2023	lung	Isolate	0.3	**87.2**	5.6	29592
A/Saint-Petersburg/182-b11V/2023	bronchi	Isolate	28.8	**65.1**	-	40862
A/Pskov/231-l35V/2023	lung	Isolate	8.9	**83.9**	-	27700
A/Kaliningrad/243-ls14V/2023	lung	Isolate	0.2	**95.6**	-	13582
A/Krasnoyarsk/230-ll30V/2022	lung	Original	37.3	**59.6**	0.3	1657
A/Khakasia/257-ll8V/2023	lung	Original	24.6	**51.8**	22.6	38580
A/Tomsk/299-l13V/2023	lung	Original	0.9	**98**	-	17758
A/KhMAO/272-l41V/2023	lung	Original	23.4	**52.1**	23.8	1314
A/Russia/319-l41V/2023	lung	Original	7.6	**76.3**	15.0	3199
A/Chelyabinsk/322-l20V/2023	lung	Original	-	**99**	0.2	4991
A/Russia/319-l40V/2023	lung	Original	0.2	**98.3**	-	49440
A/Chelyabinsk/307-t17V/2023	lung	Isolate	5.7	**94.3**	-	194
A/Novosibirsk/218-sec27V/2022	autopsy material	Isolate	1.9	20.3	**72.9**	32986
A/KHMAO/207-t22V/2022	trachea	Isolate	0.1	-	**94.2**	20166
Virus from case with recovery						
A/Orenburg/204-2V/2022	Nasopha-ryngeal swab	Isolate	0.4	**98.6**	-	49672

The table shows the percentage of virus variants with the mutations in the sample.
#-coverage is the total number of NGS sequences (reads) at amino acid position 222 in HA.

Table S7. IC_{50} of NAIs determined for isolated seasonal influenza viruses.

№	Virus	Type/subtype	Specimen collection date	Oseltamivir		Zanamivir	
				IC_{50}, nM	NA inhibition[a]	IC_{50}, nM	NA inhibition
	**Reference viruses**						
1	A/California/04/2009	A(H1N1) pdm09	-	0.20	NI	0.30	NI
2	A/North Carolina/39/2009 (H275Y)	A(H1N1) pdm09	-	113.17	HRI	0.42	NI
3	B/Phuket/3073/2013	B/Yamagata	-	9.70	NI	1.58	NI
	**Tested viruses**						
1	A/Buryatia/106-6V/2022	A/H1N1pdm09	19.11.2022	0.25	NI	0.34	NI
2	A/Yekaterinburg/111-l11V/2022	A/H1N1pdm09	21.11.2022	0.26	NI	0.38	NI
3	A/Ekaterinburg/111-l13V/2023	A/H1N1pdm09	25.11.2022	0.27	NI	0.30	NI
4	A/Krasnoyarsk/119-ll10V/2022	A/H1N1pdm09	02.12.2022	0.16	NI	0.31	NI
5	A/Yaroslavl/128-pl1V/2022	A/H1N1pdm09	06.12.2022	0.25	NI	0.38	NI
6	A/HMAO/133-lp5V/2023	A/H1N1pdm09	30.11.2022	0.25	NI	0.38	NI
7	A/Omsk/138-l7V/2022	A/H1N1pdm09	06.12.2022	0.22	NI	0.38	NI
8	A/Ulyanovsk/140-1V/2022	A/H1N1pdm09	06.12.2022	0.19	NI	0.33	NI
9	A/Gornoaltaisk/144-3V/2022	A/H1N1pdm09	01.12.2022	0.25	NI	0.33	NI
10	A/Kurgan/145-9V/2022	A/H1N1pdm09	30.11.2022	0.20	NI	0.28	NI
11	A/Kemerovo/154-10V/2022	A/H1N1pdm09	07.12.2022	0.17	NI	0.27	NI
12	A/Kemerovo/154-12V/2022	A/H1N1pdm09	07.12.2022	0.22	NI	0.23	NI
13	A/Kemerovo/154-7V/2022	A/H1N1pdm09	05.12.2022	0.22	NI	0.28	NI
14	A/Kemerovo/154-9V/2022	A/H1N1pdm09	07.12.2022	0.21	NI	0.30	NI
15	A/Vladimir/163-l2V/2022	A/H1N1pdm09	05.12.2022	0.18	NI	0.24	NI
16	A/Sakha/164-t7V/2023	A/H1N1pdm09	12.12.2022	0.24	NI	0.34	NI
17	A/Vologda/168-l8V/2023	A/H1N1pdm09	06.12.2022	0.46	NI	0.51	NI
18	A/Tatarstan/174-ll13V/2022	A/H1N1pdm09	08.12.2022	0.38	NI	0.63	NI

19	A/KhMAO/179-15V/2022	A/H1N1pdm09	13.12.2022	0.22	NI	0.37	NI
20	A/Saratov/181-11V/2022	A/H1N1pdm09	13.12.2022	0.21	NI	0.26	NI
21	A/Saratov/181-12V/2022	A/H1N1pdm09	13.12.2022	0.21	NI	0.28	NI
22	A/Russia/183-9V/2022	A/H1N1pdm09	15.12.2022	0.26	NI	0.42	NI
23	A/Tyumen/185-l13V/2022	A/H1N1pdm09	15.12.2022	0.34	NI	0.41	NI
24	A/Vologda/189-t10V/2022	A/H1N1pdm09	13.12.2022	0.23	NI	0.34	NI
25	A/Novosibirsk/192-t21V/2022	A/H1N1pdm09	19.12.2022	0.23	NI	0.31	NI
26	A/Altai Krai/193-l7V/2022	A/H1N1pdm09	20.12.2022	0.31	NI	0.36	NI
27	A/Murmansk/197-l6V/2022	A/H1N1pdm09	19.12.2022	0.23	NI	0.28	NI
28	A/Novosibirsk/198-t22V/2022	A/H1N1pdm09	21.12.2022	0.24	NI	0.31	NI
29	A/Krasnoyrsk/201-22V/2022	A/H1N1pdm09	27.12.2022	0.35	NI	0.36	NI
30	A/Krasnoyarsk/201-24V/2022	A/H1N1pdm09	21.12.2022	0.25	NI	0.32	NI
31	A/Krasnoyarsk/201-t27V/2022	A/H1N1pdm09	19.12.2022	0.28	NI	0.29	NI
32	A/Omsk/203-l8V/2022	A/H1N1pdm09	22.12.2022	0.25	NI	0.37	NI
33	A/Orenburg/204-2V/2022	A/H1N1pdm09	28.12.2022	0.25	NI	0.30	NI
34	A/Orenburg/204-4V/2022	A/H1N1pdm09	21.12.2022	0.27	NI	0.36	NI
35	A/Orenburg/204-7V/2022	A/H1N1pdm09	22.12.2022	0.23	NI	0.32	NI
36	A/HMAO/207-l23V/2022	A/H1N1pdm09	29.12.2022	0.16	NI	0.26	NI
37	A/HMAO/207-20V/2023	A/H1N1pdm09	10.12.2022	0.26	NI	0.31	NI
38	A/HMAO/207-b24V/2022	A/H1N1pdm09	29.12.2022	0.85	NI	2.34	NI
39	A/KHMAO/207-t22V/2022	A/H1N1pdm09	16.12.2022	0.28	NI	0.39	NI
40	A/Sakhalin/210-ll58V/2022	A/H1N1pdm09	29.12.2022	0.22	NI	0.34	NI
41	A/Krasnoyarsk/213am28V/2022	A/H1N1pdm09	18.12.2022	0.25	NI	0.34	NI
42	A/Kaliningrad/217-lps10V/2022	A/H1N1pdm09	21.12.2022	0.19	NI	0.33	NI
43	A/Novosibirsk/218-sec27V/2022	A/H1N1pdm09	09.12.2022	0.15	NI	0.29	NI
44	A/Ekaterinburg/225-17V/2022	A/H1N1pdm09	20.12.2022	0.21	NI	0.32	NI
45	A/Krasnoyarsk/230-ll31V/2023	A/H1N1pdm09	29.12.2022	0.27	NI	0.30	NI
46	A/Pskov/231-l35V/2023	A/H1N1pdm09	26.12.2022	0.25	NI	0.29	NI

47	A/Pskov/231-l36V/2023	A/H1N1pdm09	26.12.2022	0.27	NI	0.36	NI
48	A/Stavropol/233-l9V/2023	A/H1N1pdm09	18.12.2022	0.17	NI	0.29	NI
49	A/Irkutsk/236-l15V/2023	A/H1N1pdm09	28.12.2022	0.28	NI	0.36	NI
50	A/Yaroslavl/238-l11V/2023	A/H1N1pdm09	24.12.2022	0.28	NI	0.39	NI
51	A/Tomsk/240-l7V/2023	A/H1N1pdm09	12.01.2023	0.23	NI	0.31	NI
52	A/Kaliningrad/243-ls14V/2023	A/H1N1pdm09	23.12.2022	0.22	NI	0.28	NI
53	A/Kaliningrad/243-ls15V/2023	A/H1N1pdm09	27.12.2022	0.29	NI	0.33	NI
54	A/Saratov/250-rl14V/2023	A/H1N1pdm09	12.01.2023	0.20	NI	0.28	NI
55	A/Kaliningrad/252-tb19V/2023	A/H1N1pdm09	10.01.2023	0.24	NI	0.28	NI
56	A/Krasnoyarsk/253-ll36V/2023	A/H1N1pdm09	10.01.2023	0.20	NI	0.24	NI
57	A/KhMAO/271-39V/2023	A/H1N1pdm09	01.02.2023	0.12	NI	0.28	NI
58	A/Ulyanovsk/280-3V/2023	A/H1N1pdm09	18.01.2023	0.15	NI	0.26	NI
59	A/Ulyanovsk/280-4V/2023	A/H1N1pdm09	18.01.2023	0.20	NI	0.29	NI
60	A/KhMAO/284-45V/2023	A/H1N1pdm09	30.01.2023	0.23	NI	0.40	NI
61	A/Omsk/295-t12V/2023	A/H1N1pdm09	15.02.2023	0.22	NI	0.31	NI
62	A/Yarosavl/301-l12V/2023	A/H1N1pdm09	18.02.2023	0.25	NI	0.35	NI
63	A/Yarosavl/301-t14V/2023	A/H1N1pdm09	18.02.2023	0.22	NI	0.31	NI
64	A/Yaroslavl/301-l16V/2023	A/H1N1pdm09	18.02.2023	0.24	NI	0.31	NI
65	A/Chelyabinsk/307-t17V/2023	A/H1N1pdm09	08.01.2023	0.19	NI	0.29	NI
66	A/Russia/319-l41V/2023	A/H1N1pdm09	18.01.2023	0.19	NI	0.30	NI
67	A/Chelyabinsk/322-l20V/2023	A/H1N1pdm09	13.01.2023	0.16	NI	0.28	NI
68	A/Omsk/337-l14V/2023	A/H1N1pdm09	11.04.2023	0.19	NI	0.28	NI
69	A/Novosibirsk/57-8V/2022	A/H1N1pdm09	10.11.2022	0.22	NI	0.36	NI
70	A/NizhnyNovgorod/67-3V/2022	A/H1N1pdm09	15.11.2022	0.22	NI	0.35	NI
71	A/Novosibirsk/69-12V/2022	A/H1N1pdm09	14.11.2022	0.25	NI	0.36	NI
72	A/Belgorod/73-3V/2022	A/H1N1pdm09	11.11.2022	0.21	NI	0.36	NI
73	A/Belgorod/73-4V/2022	A/H1N1pdm09	15.11.2022	0.27	NI	0.39	NI
74	A/Belgorod/73-6V/2022	A/H1N1pdm09	11.11.2022	0.22	NI	0.35	NI

75	A/Kurgan/76-2V/2022	A/H1N1pdm09	11.11.2022	0.23	NI	0.33	NI
76	A/Chita/77-1V/2022	A/H1N1pdm09	12.11.2022	0.20	NI	0.27	NI
77	A/Chita/77-2V/2022	A/H1N1pdm09	16.11.2022	0.19	NI	0.29	NI
78	A/Chita/77-4V/2022	A/H1N1pdm09	14.11.2022	0.18	NI	0.32	NI
79	A/Rostov/79-6V/2022	A/H1N1pdm09	18.11.2022	0.23	NI	0.33	NI
80	A/Yekaterinburg/85-3V/2022	A/H1N1pdm09	17.11.2022	0.21	NI	0.35	NI
81	A/Yekaterinburg/85-4V/2022	A/H1N1pdm09	21.11.2022	0.18	NI	0.37	NI
82	A/Krasnoyarsk/87-1V/2022	A/H1N1pdm09	17.11.2022	0.29	NI	0.36	NI
83	A/Krasnoyarsk/87-6V/2022	A/H1N1pdm09	19.11.2022	0.21	NI	0.37	NI
84	A/Novosibirsk/89-15V/2022	A/H1N1pdm09	19.11.2022	0.26	NI	0.30	NI
85	A/Khabarovsk/95-10V/2022	A/H1N1pdm09	28.11.2022	0.21	NI	0.32	NI
86	A/Khabarovsk/95-6V/2022	A/H1N1pdm09	23.11.2022	0.19	NI	0.28	NI
87	A/Khabarovsk/95-7V/2022	A/H1N1pdm09	24.11.2022	0.21	NI	0.29	NI
88	A/Khabarovsk/95-9V/2022	A/H1N1pdm09	23.11.2022	0.22	NI	0.28	NI
89	A/Kamchatka/99-3V/2022	A/H1N1pdm09	29.11.2022	0.13	NI	0.29	NI
90	A/Cheboksary/293-t2V/2023	A/H1N1pdm09	13.02.2023	9.92	RI	3.37	RI
91	A/Khabarovsk/95-5V/2022	A/H3N2	21.11.2022	0.18	NI	1.00	NI
92	B/Vologda/102-5V/2022	B/Victoria	18.11.2022	16.05	NI	2.83	NI
93	B/Yekaterinburg/281-20V/2023	B/Victoria	25.01.2023	17.14	NI	3.71	NI
94	B/Perm/286-b22V/2023	B/Victoria	03.02.2023	17.83	NI	5.34	NI
95	B/Tyumen/288-24V/2023	B/Victoria	02.02.2023	16.53	NI	4.09	NI
96	B/Tyumen/288-25V/2023	B/Victoria	09.02.2023	17.31	NI	3.70	NI
97	B/Perm/302-urt26V/2023	B/Victoria	13.02.2023	16.02	NI	2.74	NI
98	B/Perm/310-b27V/2023	B/Victoria	01.03.2023	20.36	NI	3.77	NI
99	B/Lipetsk/316-1.1V/2023	B/Victoria	09.03.2023	19.85	NI	4.35	NI
100	B/KhMAO/324-63V/2023	B/Victoria	18.03.2023	19.60	NI	4.43	NI
101	B/KhMAO/325-64V/2023	B/Victoria	20.03.2023	17.35	NI	3.59	NI
102	B/KhMAO/325-65V/2023	B/Victoria	20.03.2023	15.81	NI	3.86	NI

103	B/KhMAO/328-71V/2023	B/Victoria	29.03.2023	20.37	NI	4.60	NI
104	B/KhMAO/329-76V/2023	B/Victoria	24.03.2023	15.94	NI	3.49	NI
105	B/Kalmykia/330-l53V/2023	B/Victoria	27.03.2023	17.10	NI	3.16	NI
106	B/JAO/331-ll5V/2023	B/Victoria	04.04.2023	17.00	NI	3.82	NI
107	B/KhMAO/339-78V/2023	B/Victoria	10.04.2023	18.14	NI	4.37	NI
108	B/Omsk/54-1V/2022	B/Victoria	15.11.2022	18.52	NI	3.53	NI

[a] – neuraminidase inhibition by neuraminidase inhibitors according to the WHO antiviral working group criteria: NI – normal inhibition (IC50 fold change compared to the subtype median <10 for Influenza A viruses or <5 for influenza B viruses); RI – reduced inhibition (IC50 fold change compared to the subtype median: 10-100 for Influenza A viruses or 5-50 for influenza B viruses); HRI – highly reduced inhibition (IC50 fold change compared to the subtype median: >100 for Influenza A viruses or >50 for influenza B viruses).
